# Impact of health risk factors on healthcare resource utilization, work-related outcomes and health-related quality of life of Australians: a population-based longitudinal data analysis

**DOI:** 10.3389/fpubh.2023.1077793

**Published:** 2023-11-27

**Authors:** Jun Mi, Marie Ishida, Kanya Anindya, Barbara McPake, Bernadette Fitzgibbon, Anthony A. Laverty, An Tran-Duy, John Tayu Lee

**Affiliations:** ^1^Nossal Institute for Global Health, Melbourne School of Population and Global Health, University of Melbourne, Melbourne, VIC, Australia; ^2^School of Public Health and Community Medicine, University of Gothenburg, Gothenburg, Sweden; ^3^Centre for Health Policy, Melbourne School of Population and Global Health, University of Melbourne, Melbourne, VIC, Australia; ^4^School of Public Health and Preventive Medicine, Monash University, Melbourne, VIC, Australia; ^5^Centre for Health Policy, Melbourne School of Population and Global Health, University of Melbourne, Melbourne, VIC, Australia; ^6^Australian Centre for Accelerating Diabetes Innovations (ACADI), Melbourne Medical School, University of Melbourne, Melbourne, VIC, Australia; ^7^Department of Primary Care and Public Health, School of Public Health, Faculty of Medicine, Imperial College London, London, United Kingdom

**Keywords:** smoking, alcohol consumption, physical inactivity, BMI, healthcare resource utilization, work-related outcomes, health-related quality-of-life

## Abstract

**Background:**

Health risk factors, including smoking, excessive alcohol consumption, overweight, obesity, and insufficient physical activity, are major contributors to many poor health conditions. This study aimed to assess the impact of health risk factors on healthcare resource utilization, work-related outcomes and health-related quality of life (HRQoL) in Australia.

**Methods:**

We used two waves of the nationally representative Household, Income, and Labor Dynamics in Australia (HILDA) Survey from 2013 and 2017 for the analysis. Healthcare resource utilization included outpatient visits, hospitalisations, and prescribed medication use. Work-related outcomes were assessed through employment status and sick leave. HRQoL was assessed using the SF-6D scores. Generalized estimating equation (GEE) with logit or log link function and random-effects regression models were used to analyse the longitudinal data on the relationship between health risk factors and the outcomes. The models were adjusted for age, sex, marital status, education background, employment status, equilibrium household income, residential area, country of birth, indigenous status, and socio-economic status.

**Results:**

After adjusting for all other health risk factors covariates, physical inactivity had the greatest impact on healthcare resource utilization, work-related outcomes, and HRQoL. Physical inactivity increased the likelihood of outpatient visits (AOR = 1.60, 95% CI = 1.45, 1.76 *p* < 0.001), hospitalization (AOR = 1.83, 95% CI = 1.66–2.01, *p* < 0.001), and the probability of taking sick leave (AOR = 1.31, 95% CI = 1.21–1.41, *p* < 0.001), and decreased the odds of having an above population median HRQoL (AOR = 0.48, 95% CI = 0.45–0.51, *p* < 0.001) after adjusting for all other health risk factors and covariates. Obesity had the greatest impact on medication use (AOR = 2.02, 95% CI = 1.97–2.29, *p* < 0.001) after adjusting for all other health risk factors and covariates.

**Conclusion:**

Our study contributed to the growing body of literature on the relative impact of health risk factors for healthcare resource utilization, work-related outcomes and HRQoL. Our results suggested that public health interventions aim at improving these risk factors, particularly physical inactivity and obesity, can offer substantial benefits, not only for healthcare resource utilization but also for productivity.

## Introduction

The contour of Australia's population health is transforming, characterized by increased longevity and a growing demand for healthcare (1–7). Concurrently, the prevalence of many chronic diseases and their associated risk factors is on the rise (8, 9). The latest official Australian report showed smoking prevalence was 11.6% in 2019, accounting for 7.8% of overall burden of disease (10). Smoking not only poses direct risks but also exposes children of the smokers to the dangers of passive smoking and therefore to the toxic residue known as third hand smoke (11). Despite a decrease in the prevalence of harmful drinking from 21 to 16.8% between 2001 and 2019, it still accounted for about 4.5% of the total disease burden in 2015 (12, 13). Alcohol consumption could negatively affect on life expectancy in both low-income and high-income countries, even if it may positively impact the gross national income (14). Inadequate physical activity accounted for 2.5 to 6.6% of Australia's total disease burden (15–17). Moreover, half of the adult population did not meet the recommended daily exercise requirement of 10 minutes (18). Obesity and overweight are becoming more common. In 2017–2018, 67% of Australians were classified as overweight or obese, accounting for 8.4% of the overall disease burden (19).

Previous studies 师教培 in Australia have shown that the economic burden of tobacco smoking reached approximately $137 billion (20). Smoking might cause 2.4 million productivity-adjusted life years lost (21). Furthermore, a dose-response relationship has been observed between smoking status and the SF-6D health utility score (22). The cost of obesity was about $11.8 billion in 2018 (23). Without any intervention, this figure is projected to escalate to an estimated $87.7 billion by 2032 (23). A recent study indicated that if the incidence of obesity remains unchanged, 81 million productivity-adjusted life years (discounted) lived (24). Moreover, when compared to those with a healthy weight, the SF-6D utility score in the obese population is 4% lower (21). Additionally, alcohol misuse accounted for $2.57 billion in costs, while physical inactivity contributing to an approximate expenditure of $850 million (25).

However, the current literature lacks a comprehensive evaluation of the economic implications associated with health risk factors, for example the impact of obesity and physical inactivity on use of medications, absenteeism, and HRQoL (1–3, 24, 25). Besides, previous studies have primarily focused on certain cohorts rather than being population-based (26, 27). To fill this research gap, this study employs population-based data to investigate the effects of four common health risk factors (smoking, physical inactivity, alcohol consumption, and BMI) on healthcare utilization, work-related outcomes, and health-related quality of life (HRQoL).

## Methods

### Data and sample

We conducted a longitudinal data analysis using data from the Household, Income, and Labor Dynamics in Australia (HILDA) Survey. The HILDA Survey is a household-based study initiated in 2001 that collects information on many aspects of life such as employment status, income, personal well-beings and education. Data is collected from individuals aged 15 and above through a blend of self-administered questionnaires and structured interviews. HILDA survey is funded by the Australian Government Department of Social Services (DSS). Further information about the HILDA Survey can be found at the website of the Melbourne Institute (28).

This is a secondary data analysis. Data is managed by Australian Data Archive (ADA), which is not openly accessible. We applied for data access by sending a request to the ADA via its website (https://dataverse.ada.edu.au/dataverse/DSSLongitudinalStudies) and signing a Confidentiality Deed Poll with the ADA and the Department of Social Service (DSS). Access to the HILDA General Release 17 (waves 1–17) was granted in November 2019.

**Figure 1:**
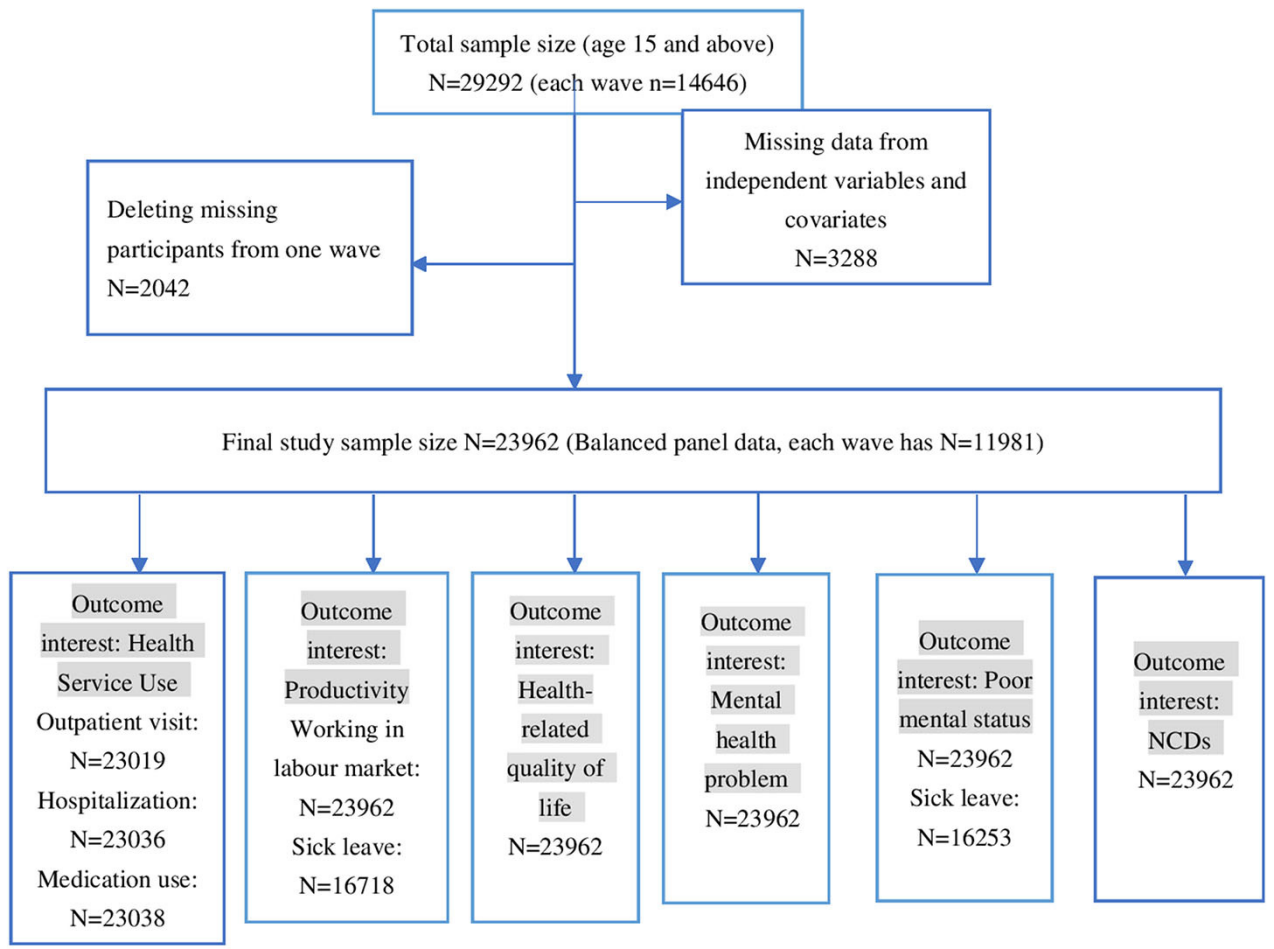
Relative impact of risk factors on outpatient visit. ^†^GEE AOR is Model 1: population average model with logit link function. ^††^Random effect (RE) is Model 2: RE logistic model. ^†††^GEE Coefficients is Model 3: population average model. ^††*††*^RE Coefficients is Model 4: is RE linear model. ^††*†††*^AOR is adjusted odds ratio.

This study included 14,646 respondents from both wave 13 (conducted in 2013) and wave 17 (conducted in 2017) as the variables of interest in our study are assessed on a quadrennial basis. After eliminating entries with incomplete data on the study variables and associated covariates, the final analysis comprised 11,981 distinct respondents. Figure 1 shows the study flow chart.

### Explanatory variables

This study examined four common health risk factors, including smoking, alcohol consumption, physical inactivity, and BMI. Respondents were categorized into different levels of exposure to health risk factors based on HILDA questionnaire: tobacco smoking was categorized as non-smoker (reference group), ex-smoker, current smoker (including smoke at least weekly, smoke less often than weekly and, smoker daily and above). Alcohol consumption was classified as no alcohol intake (reference group), former drinker, low-frequency alcohol intake (drinking alcohol for 1–2 days per week), moderate-frequency alcohol intake (drinking alcohol for 2–4 days per week), high-frequency alcohol intake (drinking alcohol for 5–6 days or everyday per week). Frequency of physical activity was classified into three levels according to the International Physical Activity Questionnaire (IPAQ), high-level physical activity (reference group, at least 1,500 MET-minutes per week), moderate-level physical activity (at least 600 MET-minutes per week) and low-level physical activity (not meet any criteria). BMI was classified as underweight (BMI less than 18.5 kg/m2), normal BMI (BMI between 18.5 kg/m^2 − 2^4.9 kg/m^2^) overweight (BMI between 25.0–29.9 kg/m2), and obese (BMI >30 kg/m2).

The common explanatory variables for all outcomes are: age (15–24, 25–34, 35–44, 45–54, 55–64, 65–74, 75 and above), sex (male, female), education background (less than high school or equivalent, high school or equivalent, bachelor and above), employment status (employed and not employed), country of birth (born in Australia, born in main English speaking country, and other), state (New South Wales, Victoria, Queensland, South Australia, West Australia, Tasmania, Northern Territory and Australian Capital Territory), residency (urban or rural), Indigenous status (non-Indigenous, Australians and Indigenous Australian), marital status (married or defacto, unmarried, separated or divorced or widowed), quintiles of neighborhood socio-economic status measured by socio-economic indexed for areas (SEIFA), and quintiles of equilibrium family income (29). Both SEIFA and equilibrium family income use 1^st^ quintile as reference group. In the analysis focused on work-related outcomes, employment status was not incorporated as an explanatory variable. In examining the relationship between sick leave and health risk factors, the analysis was conducted only for people with a paid job.

### Outcome variables

This study considers the healthcare resource utilization, work-related outcomes, and HRQoL as outcome interests based on the WHO's recommendation for cost- of- illness (30, 31). The healthcare resource utilization included in this study were: (1) any outpatient visits in the past 12 months (binary outcome with 0 is no visits, and 1 is at least one visit in the last 12 months); (2) number of outpatient visits in the past 12 months; (3) any hospitalization in the past 12 months (binary outcome with 0 is no stay, and 1 is at least one night stay in the last 12 months); (4) number of nights spent in hospital admission; (5) use of any prescribed medication in the past 12 months (binary outcome with 0 is not taken any medication and 1 is taken at least one prescribed medication); The following work-related outcomes were: (1) whether respondents were employed (binary outcome with 0 is not employed for the last 12 months, and 1 is employed in the last 12 months); (2) whether respondents have taken at least 1 day sick leave in the last 12 months); (3) how many days of sick leave have they taken in the past 12 months. HRQoL was measured using SF-6D and expressed as binary outcome with 0 indicating a value below sample the median SF-6D score, and 1 indicating a value equal or above the median SF-6D score (32). SF-6D is a composite metric that includes physical functioning, role limitation, social functioning, pain, mental health, and vitality, ranging from 0 (lowest) to 1 (highest) (33).

### Statistical analysis

The relative impact of health risk factors on healthcare and economic outcomes were investigated using longitudinal study design. First, we used a series of generalized estimating equations (GEE) to estimate the effects of risk factors on outcomes. The GEE model estimated the population-average effects of risk factors, and the model used the identify, logit link functions for binary outcome variables (such as any outpatient visits), and log link functions for continuous outcome variables (such as the number of days in hospital). Second, we employed random effect models to examine the same outcomes that can provide more robust and comprehensive results by accounting for different sources of variation and bias. The random effects model is a statistical tool that accounts for significant intra-individual heterogeneity that may persist over time. The random effects model is particularly useful in longitudinal studies where multiple measurements are taken from the same entities over time. Using both models can help to identify potential differences and similarities in the estimated effects of health risk factors, and to provide more complete and reliable results. We used robust standard error in both the GEE and random effect models. *P-*values < 0.05 were considered statistically significant. All analyses were conducted using Stata 16 (Stata Corp).

## Results

### Sample characteristics

Sample characteristics were presented in Table 1. The median age in wave 17 was 49 years old (IQR 34–63), 53.9% of the sample were female and the majority were married (67.5%), 34.2% were overweight and 26.3% were obese, 31.0% reported low physical activity, and 14.6% reported high-frequency alcohol intake. 29.7% were former smokers and 15.2% were current smokers.

**Table 1:** Sample characteristics from longitudinal samples of HILDA wave 17.

**Variables**	**Frequency (%)**
**Sex**	
Male	5,526 (46.1)
Female	6,455 (53.9)
**Age**	
15–24	1,078 (9.0)
25–34	2,026 (16.9)
35–44	1,890 (15.8)
45–54	2,145 (17.9)
55–64	2,130 (17.8)
65–74	1,631 (13.6)
75 and above	1,081 (9.0)
**Education level**	
Less than senior secondary	2,624 (21.9)
Equal to secondary	5,852 (48.8)
Bachelor and above	3,505 (29.3)
**Employment status**	
Employed	7,613 (63.5)
Unemployed	4,368 (36.5)
**Equilibrium family income**	
Lowest quintile	2,857 (19.5)
1st quintile	2,938 (20.1)
2nd quintile	2,941 (20.1)
3rd quintile	2,962 (20.2)
Highest quintile	2,948 (20.1)
**Marital status**	
Married/defacto	8,092 (67.5)
Unmarried	2,132 (17.8)
Separated/divorce/widowed	1,757 (14.7)
**Residential area**	
Urban	10.397 (86.8)
Rural	1,584 (13.2)
**Residential state**	
NSW	3,486 (29.1)
VIC	2,929 (24.5)
QLD	2,566 (21.4)
SA	1,100 (9.2)
WA	1,131 (9.4)
Tasmania	412 (3.4)
Northern Territory	96 (0.8)
ACT	261 (2.2)
**Country of birth**	
Australia	9,433 (78.7)
English speaking country	1,187 (9.9)
Other	1,361 (11.4)
**Aboriginal status**	
Non-aboriginal	11,734 (98.0)
Aboriginal	247 (2.1)
**Socio-economic indices for areas (SEIFA)**	
1st and 2nd decline	2,160 (18.0)
3rd and 4th decline	2,376 (19.8)
5th and 6th decline	2,366 (19.8)
7th and 8th decline	2,540 (21.2)
9th and 10th decline	2,539 (21.2)
**Body mass index (BMI)**	
Normal	4,129 (34.5)
Underweight	598 (5.0)
Overweight	4,102 (34.2)
Obese	3,152 (26.3)
**Physical activity**	
Low	3,708 (31.0)
Moderate	4,122 (34.4)
High	4,151 (34.7)
**Alcohol intake**	
No or rare intake	3,847 (32.1)
No longer intake	1,085 (9.1)
Low frequency intake	2,253 (18.8)
Moderate frequency intake	3,045 (25.4)
High frequency intake	1,751 (14.6)
**Smoking status**	
Non-smoker	6,603 (55.1)
Ex-smoker	3,552 (29.7)
Current smoker	1,826 (15.2)

### The relative impact of risk factors on healthcare resource utilization

#### Outpatient visits

Based on the results from Model 1, insufficient physical activity was identified as an important risk factor after adjusting for all other health risk factors and covariates. Specifically, low-level of physical activity (AOR = 1.60, 95% CI = 1.45–1.76, *p* < 0.001) was associated with the highest probability of outpatient visits compared to high-level of physical activity after adjusting for all other health risk factors and covariates in Model 1 based on GEE logit function. Furthermore, being an ex-smoker was associated with a higher risk of using healthcare resource compared to a non-smoker (AOR = 1.41, 95% CI = 1.28–1.56, p < 0.001) after adjusting for all other health risk factors and covariates. Obesity was also associated with a higher likelihood of outpatient visits (AOR = 1.36, 95% CI = 1.23–1.52, p < 0.001) when compared with normal BMI. These observations were in line with Model 2.

**Figure 2:**
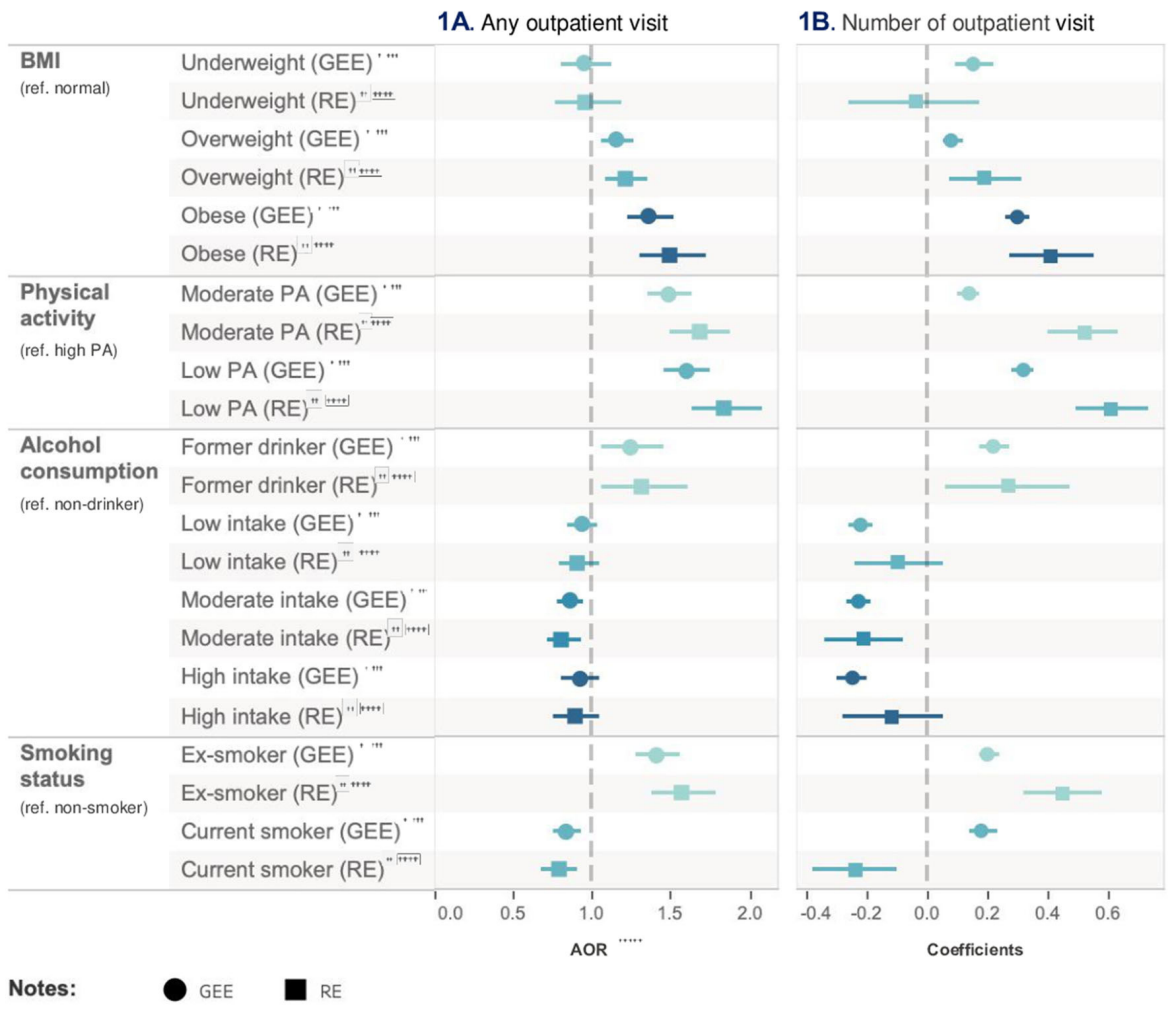
Relative impact of risk factors on hospitalization. ^†^GEE AOR is Model 1: population average model with logit link function. ^††^Random effect (RE) is Model 2: RE logistic model. ^†††^GEE Coefficients is Model 3: population average model. ^††*††*^RE Coefficients is Model 4: is RE linear model. ^††*†††*^AOR is adjusted odds ratio.

Based on the GEE log function, after adjusting for all other risk factors and covariates, Model 3 showed that the estimated mean of outpatient visits in days was higher in people with low-level of physical activity (coef = 0.32, 95% CI = 0.28–0.35, p < 0.001) and moderate-level physical activity (coef = 0.14, 95% CI = 0.10, 0.17 *p* < 0.001) compared to those with high-level of physical activity. When compared to non-smoker, being former smokers (coef = 0.20, 95% CI = 0.17–0.24, p < 0.001) also increased the estimated mean of outpatient visits. Moderate-frequency alcohol and former drinking were associated with lower (coef = −0.23, 95% CI = −0.19–0.27, *p* < 0.001) and higher estimated mean of outpatient visits (coef = 0.22, 95% CI = 0.17–0.27, *p* < 0.001) compared to no drinking. Model 4, the random effect model, produced similar results as those by Model 3. Table 2 and Figure 2 show the relative impact of risk factors on outpatient visit. Table 2a shows the unadjusted model results.

**Table 2:** Relative impact of risk factors on healthcare resource use (outpatient visit).

**Risk factors**	**Any outpatient visit**		**Number of outpatient visit**	
	**Model 1** ^†^	**Model 2** ^††^	**Model 3** ^†††^	**Model 4** ^††*††*^
	**AOR**^††*†††*^ **(*****P-*****value) (95% CI)**	**AOR**^††*†††*^ **(*****P-*****value) (95% CI)**	**Coef**^††*†††*^ **(*****P-*****value) (95% CI)**	**Coef**^††*†††*^ **(*****P-*****value) (95%CI)**
**BMI**				
**Normal (ref)**				
Underweight	0.95 (0.554) (0.81 to 1.12)	0.95 (0.705) (0.77 to 1.19)	0.15 (<0.001) (0.09 to 0.22)	−0.04 (0.705) (−0.26 to 0.17)
Overweight	1.16 (0.001) (1.06 to 1.27)	1.21 (0.001) (1.08 to 1.36)	0.08 (<0.001) (0.05 to 0.12)	0.19 (0.001) (0.07 to 0.31)
Obese	1.37 (<0.001) (1.23 to 1.52)	1.50 (<0.001) (1.30 to 1.73)	0.30 (<0.001) (0.26 to 0.34)	1.50 (<0.001) (0.27 to 0.55)
**Physical activity**				
**High activity (ref)**				
Moderate activity	1.49 (<0.001) (1.36 to 1.63)	1.68 (<0.001) (1.49 to 1.88)	0.14 (<0.001) (0.10 to 0.17)	0.52 (<0.001) (0.40 to 0.63)
Low activity	1.60 (<0.001) (1.45 to 1.76)	1.84 (<0.001) (1.63 to 2.08)	0.32 (<0.001) (0.28 to 0.35)	0.61 (<0.001) (0.49 to 0.73)
**Alcohol intake**				
**Non-drinker (ref)**				
No longer drunk	1.24 (0.009) (1.06 to 1.50)	1.31 (0.012) (1.06 to 1.61)	0.22 (<0.001) (0.17 to 0.27)	0.27 (0.012) (0.06 to 0.47)
Low intake	0.94 (0.235) (0.84 to 1.04)	0.91 (0.188) (0.79 to 1.05)	−0.22 (<0.001) (−0.26 to −0.18)	−0.09 (0.188) (−0.24 to 0.05)
Moderate intake	0.86 (0.004) (0.78 to 0.95)	0.81 (0.002) (0.71 to 0.93)	−0.23 (<0.001) (−0.27 to −0.19)	−0.21 (0.002) (−0.34 to −0.08)
High intake	0.92 (0.197) (0.81 to 1.05)	0.89 (0.163) (0.75 to 1.05)	−0.25 (<0.001) (−0.30 to −0.20)	−0.12 (0.163) (−0.28 to −0.05)
**Smoking status**				
**Non-smoker (ref)**				
Ex-smoker	1.41 (<0.001) (1.28 to 1.56)	1.57 (<0.001) (1.38 to 1.79)	0.20 (<0.001) (0.17 to 0.24)	0.45 (<0.001) (0.32 to 0.58)
Current smoker	0.84 (0.001) (0.75 to 0.93)	0.79 (0.001) (0.68 to 0.91)	0.18 (<0.001) (0.14 to 0.23)	−0.24 (0.001) (−0.38 to −0.10)

^†^Model 1 is GEE population average model with logit link function.

^††^Model 2 is random effect logistic model.

^†††^Model 3 is GEE population average model.

^††*††*^Model 4 is random effect linear model.

^††*†††*^AOR, adjusted odds ratio; Coef, coefficient; 95% CI, 95% confidence interval.

**Table 2a:** Unadjusted regression models for impact of risk factors on healthcare resource utilization (outpatient visit).

**Risk factors**	**Any outpatient visit**	**Number of outpatient visit**
	**Model 1** ^†^	**Model 2** ^††^
	**AOR**^†††^ **(*****P-*****value) (95% CI)**	**Coef**^†††^ **(*****P-*****value) 95% CI**
**BMI**		
**Normal (ref)**		
Underweight	1.01 (0.927) (0.86 to 1.18)	0.22 (<0.001) (0.16 to 0.29)
Overweight	1.20 (<0.001) (1.10 to 1.32)	0.10 (<0.001) (0.07 to 0.14)
Obese	1.51 (<0.001) (1.36 to 1.68)	0.38 (<0.001) (0.34 to 0.42)
**Physical activity**		
**High activity (ref)**		
Moderate activity	1.53 (<0.001) (1.40 to 1.67)	0.15 (<0.001) (0.12 to 0.19)
Low activity	1.68 (<0.001) (1.53 to 1.84)	0.38 (<0.001) 0.34 to 0.41)
**Alcohol intake**		
**Non-drinker (ref)**		
No longer drunk	1.32 (0.001) (1.13 to 1.56)	0.27 (<0.001) (0.22 to 0.33)
Low intake	0.91 (0.092) (0.81 to 1.02)	−0.24 (<0.001) (−0.28 to −0.20)
Moderate intake	0.86 (0.004) (0.78 to 0.95)	−0.24 (<0.001) (−0.28 to −0.20)
High intake	0.96 (0.496) (0.84 to 1.09)	−0.24 (<0.001) (−0.29 to −0.19)
**Smoking status**		
**Non-smoker (ref)**		
Ex-smoker	1.46 (<0.001) (1.32 to 1.61)	0.22 (<0.001) (0.18 to 0.25)
Current smoker	0.83 (0.001) (0.75 to 0.92)	0.18 (<0.001) (0.13 to 0.22)

^†^Model 1 is unadjusted GEE population average model with logit link function.

^††^Model 2 is unadjusted GEE population average model.

^†††^OR, unadjusted odds ratio; Coef, unadjusted coefficient; 95% CI, 95% confidence interval.

#### Hospitalization

In Model 1, low-level physical activity had an 83% higher likelihood of being hospitalized compared with high-level physical activity after adjusting for all other health risk factors and covariates (95% CI = 1.66–2.01, *p* < 0.001). Compared with non-smoker, ex-smoker (AOR = 1.46, 95% CI = 1.33–1.60, *p* < 0.001) increased the likelihood on hospitalization. Moderate-frequency alcohol user after adjusting for all other health risk factors and covariates (AOR = 0.75, 95% CI = 0.68–0.83, *p* < 0.001) was less likely to be hospitalized than non-drinkers. Being a former drinker, on the other hand, was associated with an increased likelihood of hospitalization (AOR = 1.36, 95% CI = 1.19, 1.55, *p* < 0.001). Results from Model 2 were consistent with Model 1 but had a more significant effect.

**Figure 3:**
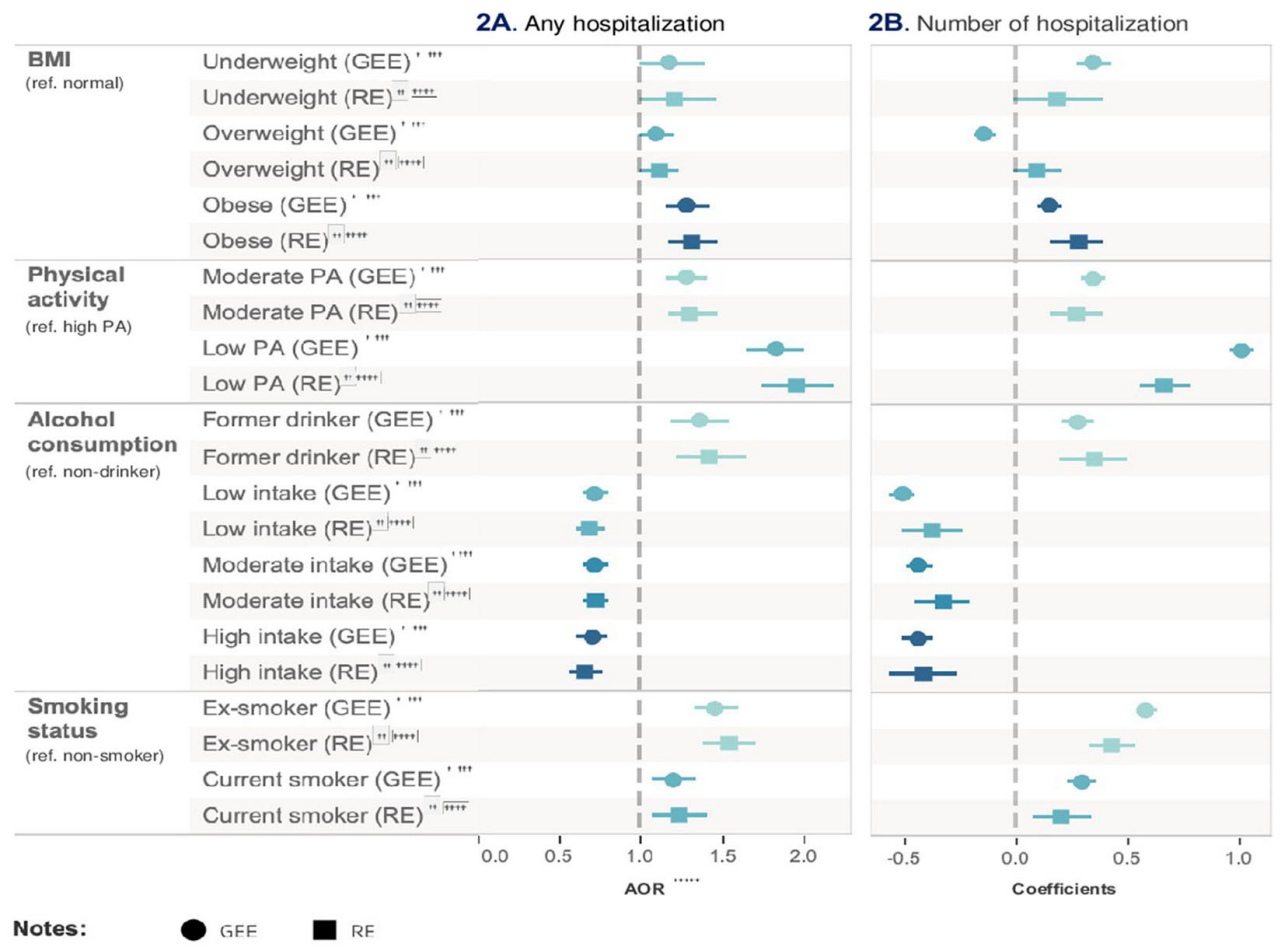
Relative impact of risk factors on medication. ^†^GEE AOR is Model 1: population average model with logit link function. ^††^Random effect (RE) is Model 2: RE logistic model. ^†††^AOR is adjusted ratio.

In Model 3, low-level physical activity (coef = 1.02, 95% CI = 0.96–1.07, *p* < 0.001), compared to high-level physical activity, remained the most important risk factor for the prolonged mean hospitalization after adjusting for all other health risk factors and covariates. Both moderate-frequency (coef = −0.44, 95% CI = −0.38 to −0.49, *p* < 0.001) and high-frequency (coef = −0.44, 95% CI = −0.38 to −0.51, *p* < 0.001) alcohol intake had the same effect on reducing the mean of hospitalization after adjusting for all other health risk factors and covariates. Results from Model 4 were consistent with Model 3. The relative impact of risk factors on hospitalization is presented in Table 3 and Figure 3. Table 3a shows the unadjusted model results.

**Table 3:** Relative impact of risk factors on healthcare resource utilization (hospitalization).

**Risk factors**	**Any hospitalization**		**Number of hospitalization (per nights)**	
	**Model 1** ^†^	**Model 2** ^††^	**Model 3** ^†††^	**Model 4** ^††*††*^
	**AOR**^††*†††*^ **(*****P-*****value) (95% CI)**	**AOR**^††*†††*^ **(*****P-*****value) (95% CI)**	**Coef**^††*†††*^ **(*****P-*****value) (95% CI)**	**Coef**^††*†††*^ **(*****P-*****value) (95% CI)**
**BMI**				
**Normal (ref)**				
Underweight	1.16 (0.068) (0.99 to 1.40)	1.21 (0.061) (0.99 to 1.47)	0.35 (<0.001) (0.27 to 0.43)	0.19 (0.061) (−0.01 to 0.39)
Overweight	1.09 (0.072) (0.99 to 1.21)	1.11 (0.075) (0.99 to 1.24)	−0.14 (<0.001) (−0.19 to −0.09)	0.10 (0.075) (−0.01 to 0.21)
Obese	1.28 (<0.001) (1.15 to 1.42)	1.32 (<0.001) (1.17 to 1.48)	0.15 (<0.001) (0.010 to 0.21)	0.28 (<0.001) (0.16 to 0.39)
**Physical activity**				
**High activity (ref)**				
Moderate activity	1.28 (<0.001) (1.16 to 1.41)	1.31 (<0.001) (1.17 to 1.47)	0.35 (<0.001) (0.29 to 0.40)	0.27 (<0.001) (0.16 to 0.39)
Low activity	1.83 (<0.001) (1.66 to 2.01)	1.96 (<0.001) (1.75 to 1.19)	1.02 (<0.001) (0.97 to 1.07)	0.67 (<0.001) (0.56 to 0.79)
**Alcohol intake**				
**Non-drinker (ref)**				
No longer drunk	1.36 (<0.001) (1.19 to 1.55)	1.42 (<0.001) (1.22 to 1.65)	0.28 (<0.001) (0.21 to 0.35)	0.35 (<0.001) (0.20 to 0.50)
Low intake	0.72 (<0.001) (0.64 to 0.81)	0.68 (<0.001) (0.60 to 0.78)	−0.51 (<0.001) (−0.57 to −0.45)	−0.38 (<0.001) (−0.51 to −0.24)
Moderate intake	0.75 (<0.001) (0.68 to 0.83)	0.72 (<0.001) (0.64 to 0.81)	−0.44 (<0.001) (−0.49 to −0.38)	−0.33 (<0.001) (−0.45 to −0.21)
High intake	0.70 (<0.001) (0.61 to 0.80)	0.66 (<0.001) (0.57 to 0.76)	−0.44 (<0.001) (−0.51 to −0.38)	−0.42 (<0.001) (−0.57 to −0.27)
**Smoking status**				
**Non-smoker (ref)**				
Ex-smoker	1.46 (<0.001) (1.33 to 1.60)	1.54 (<0.001) (1.39 to 1.71)	0.59 (<0.001) (0.55 to 0.64)	0.43 (<0.001) (0.33 to 0.54)
Current smoker	1.20 (0.002) (1.07 to 1.35)	1.24 (0.001) (1.08 to 1.41)	0.30 (<0.001) (0.24 to 0.36)	0.21 (<0.001) (0.08 to 0.34)

^†^Model 1 is GEE population average model with logit link function.

^††^Model 2 is random effect logistic model.

^†††^Model 3 is GEE population average model.

^††*††*^Model 4 is random effect linear model.

^††*†††*^AOR, adjusted odds ratio; Coef, coefficient; 95% CI, 95% confidence interval.

**Table 3a:** Unadjusted regression model for Impact of risk factors on healthcare resource utilization (hospitalization).

**Risk factors**	**Any outpatient visit**	**Number of outpatient visit (per nights)**
	**Model 1** ^†^	**Model 2** ^††^
	**AOR**^†††^ **(*****P-*****value) (95% CI)**	**Coef**^†††^ **(*****P-*****value) (95% CI)**
**BMI**		
**Normal (ref)**		
Underweight	1.33 (0.001) (1.12 to 1.57)	0.51 (<0.001) (0.43 to 0.59)
Overweight	1.14 (0.009) (1.03 to 1.25)	−0.14 (<0.001) (−0.19 to −0.09)
Obese	1.49 (<0.000) (1.35 to 1.65)	0.34 (<0.000) (1.29 to 0.39)
**Physical activity**		
**High activity (ref)**		
Moderate activity	1.31 (<0.001) (1.18 to 1.44)	0.38 (<0.001) (0.33 to 0.44)
Low activity	1.97 (<0.001) (1.79 to 2.17)	1.14 (<0.001) (1.09 to 1.19)
**Alcohol intake**		
**Non-drinker (ref)**		
No longer drunk	1.50 (<0.001) (1.32 to 1.71)	0.41 (<0.001) (0.34 to 0.47)
Low intake	0.70 (<0.001) (0.62 to 0.79)	−0.57 (<0.001) (−0.63 to −0.51)
Moderate intake	0.75 (<0.001) (0.68 to 0.83)	−0.46 (<0.001) (−0.51 to −0.40)
High intake	0.74 (<0.001) (0.65 to 0.84)	−0.38 (<0.001) (−0.44 to −0.31)
**Smoking status**		
**Non-smoker (ref)**		
Ex-smoker	1.45 (<0.001) (1.33 to 1.59)	0.56 (<0.001) (0.52 to 0.61)
Current smoker	1.19 (0.003) (1.06 to 1.34)	0.22 (<0.001) (0.16 to 0.28)

^†^Model 1 is unadjusted GEE population average model with logit link function.

^††^Model 2 is unadjusted GEE population average model.

^†††^OR, unadjusted odds ratio; Coef, coefficient; 95% CI, 95% confidence interval.

#### Any prescribed medication

**Figure 4:**
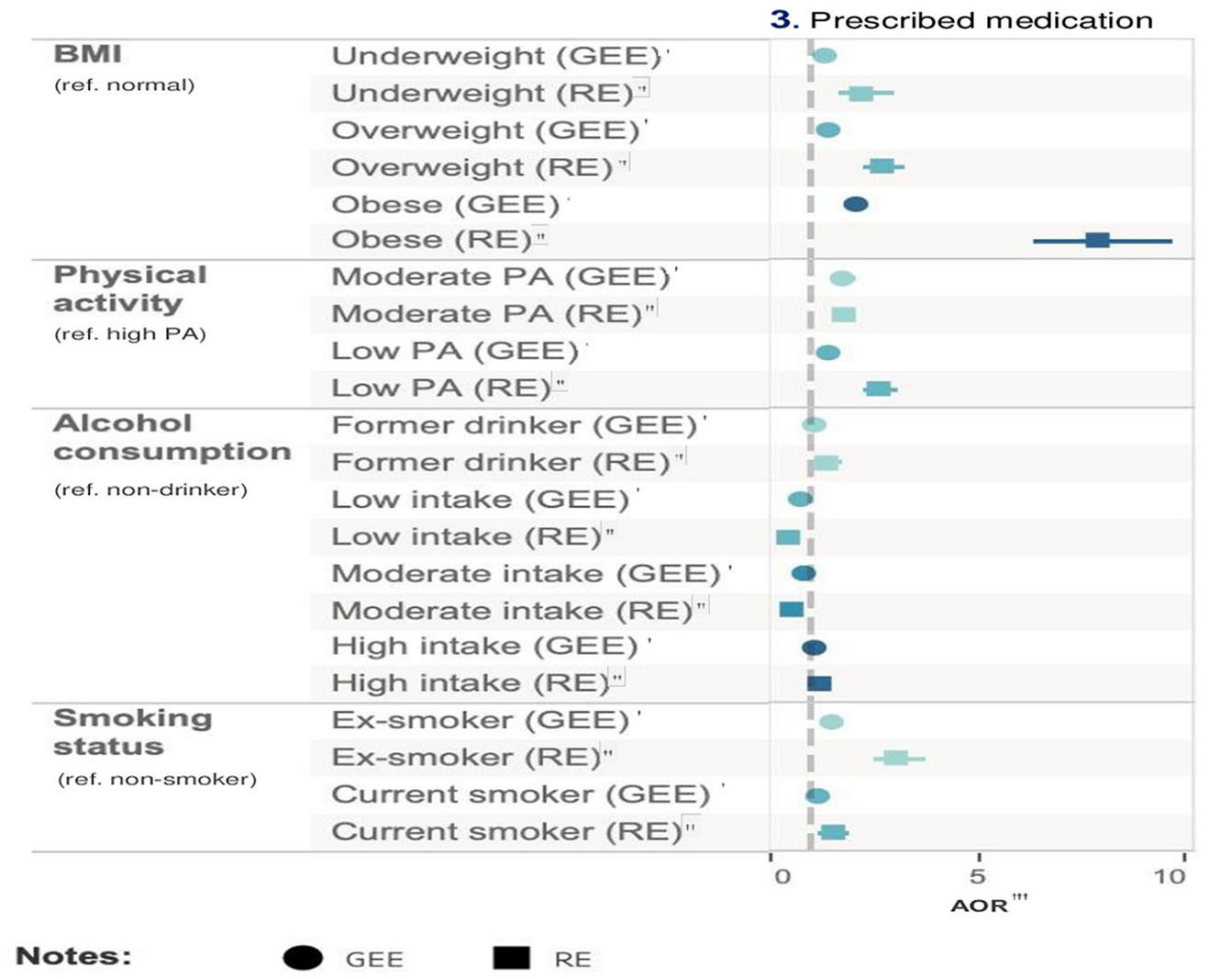
Relative impact of risk factors on labor market outcomes. ^†^GEE AOR is Model 1: population average model with logit link function. ^††^Random effect (RE) is Model 2: RE logistic model. ^†††^GEE Coefficients is Model 3: population average model. ^††*††*^RE Coefficients is Model 4: is RE linear model. ^††*†††*^AOR is adjusted odds ratio.

In Model 1, obesity was associated with an increased risk of prescribed medication use by 2.12 times (95% CI = 1.97–2.29, *p* < 0.001) compared with normal BMI participants after adjusting for all other risk factors and covariates. The next risk factor associated with greater prescribed medication use was ex-smoking (AOR = 1.50, 95% CI = 1.39–1.60, *p* < 0.001) after adjusting for all other risk factors and covariates. On the other hand, low-frequency alcohol intake (AOR = 0.77, 95% CI = 0.71–0.83, *p* < 0.001) was associated with a lower risk of medication use. The results from Model 2 were consistent with those from Model 1. As medication use is a binary variable, Models 3 and 4 were not applicable. Table 4 and Figure 4 show the relative impact of risk factors on medication. Table 4a shows the unadjusted model results.

**Table 4:** Relative impact of risk factors on healthcare resource utilization (medication).

**Risk factors**	**Prescribed medication use**	
	**Model 1** ^†^	**Model 2** ^††^
	**AOR**^†††^ **(*****P-*****value) (95% CI)**	**AOR**^†††^ **(*****P-*****value) (95% CI)**
**BMI**		
**Normal (ref)**		
Underweight	1.34 (<0.001) (1.20 to 1.49)	2.22 (<0.001) (1.66 to 2.99)
Overweight	1.44 (<0.001) (1.35 to 1.54)	2.70 (<0.001) (2.27 to 3.20)
Obese	2.12 (<0.001) (1.95 to 2.29)	7.91 (<0.001) (6.41 to 9.76)
**Physical activity**		
**High activity (ref)**		
Moderate activity	1.25 (<0.001) (1.18 to 1.32)	1.79 (<0.001) (1.53 to 2.08)
Low activity	1.45 (<0.001) (1.36 to 1.54)	2.65 (<0.001) (2.25 to 3.13)
**Alcohol intake**		
**Non-drinker (ref)**		
No longer drunk	1.13 (0.009) (0.71 to 0.83)	1.37 (0.015) (1.06 to 1.78)
Low intake	0.77 (<0.001) (1.03 to 1.23)	0.47 (<0.001) (0.38 to 0.57)
Moderate intake	0.81 (<0.001) (0.76 to 0.86)	0.54 (<0.001) (0.45 to 0.65)
High intake	1.07 (0.117) (0.98 to 1.18)	1.21 (0.124) (0.95 to 1.54)
**Smoking status**		
**Non-smoker (ref)**		
Ex-smoker	1.50 (<0.001) (1.39 to 1.60)	3.10 (<0.001) (2.55 to 3.78)
Current smoker	1.15 (0.002) (1.05 to 1.25)	1.53 (<0.001) (1.21 to 1.94)

^†^Model 1 is GEE population average model with logit link function.

^††^Model 2 is random effect logistic model.

^†††^AOR, adjusted odds ratio; Coef, coefficient; 95% CI– 95% confidence interval.

**Table 4a:** Unadjusted regression model for impact of risk factors on healthcare resource utilization (medication).

**Risk factors**	**Prescribed medication use**
	**Model 1** ^†^
	**AOR**^††^ **(*****P-*****value) (95% CI**
**BMI**	
**Normal (ref)**	
Underweight	1.38 (<0.001) (1.24–1.54)
Overweight	1.46 (<0.001) 1.37–1.56)
Obese	2.23 (<0.001) (2.07–2.40)
**Physical activity**	
**High activity (ref)**	
Moderate activity	1.27 (<0.001) (1.20–1.34)
Low activity	1.45 (<0.001) (1.42–1.59)
**Alcohol intake**	
**Non-drinker (ref)**	
No longer drunk	1.20 (<0.001) (1.10–1.31)
Low intake	0.78 (<0.001) (0.73–0.84)
Moderate intake	0.83 (<0.001) (0.78–0.89)
High intake	1.11 (0.014) (1.02–1.21)
**Smoking status**	
**Non-smoker (ref)**	
Ex-smoker	1.56 (<0.001) (1.46–1.67)
Current smoker	1.17 (<0.001) (1.08–1.28)

^†^Model 1 is unadjusted GEE population average model with logit link function.

^††^OR, unadjusted odds ratio; Coef, coefficient; 95% CI, 95% confidence interval.

### The relative impact of risk factors on work-related outcomes

#### Employment status

In Model 1, the low-level physical activity group (AOR = 0.57, 95% CI = 0.53–0.61, *p* < 0.001) was associated with a reduced likelihood of employed compared with high-level physical activity after adjusting for all other health risk factors and covariates. Alcohol consumption, particularly among low-frequency drinkers, was associated with a higher likelihood of employment (AOR = 2.04, 95% CI = 0.76–0.91, *p* < 0.001) compared to non-drinkers after adjusting for all other health risk factors and covariates. Former drinkers were associated with a reduced likelihood of employment (AOR = 0.83, 95% CI = 1.88–2.20, *p* < 0.001) compared with non-drinker after adjusting for all other risk factors and covariates. As employment status is a binary variable, Models 3 and 4 were not applicable.

#### Absenteeism

In Model 1, compared to the high-level physical activity group, individuals with low-level physical activity exhibited an increased likelihood of having sick leave (AOR = 1.31, 95% CI = 1.21–1.41, *p* < 0.001) after adjusting for all other health risk factors and covariates. Moderate-level physical activity group also increased the likelihood for taking sick leave (AOR = 1.30, 95% CI = 1.21–1.39, *p* < 0.001) compared with high-level physical activity group after adjusting for all other health risk factors and covariates. Low-frequency alcohol consumption (AOR = 1.27, 95% CI = 1.15, 1.39, *p* < 0.001) and obesity (AOR = 1.26, 95%CI = 1.15, 1.38, *p* < 0.001) had a similar impact on increasing absenteeism after adjusting for all other health risk factors and covariates. Model 2 showed similar results to Model 1.

**Figure 5:**
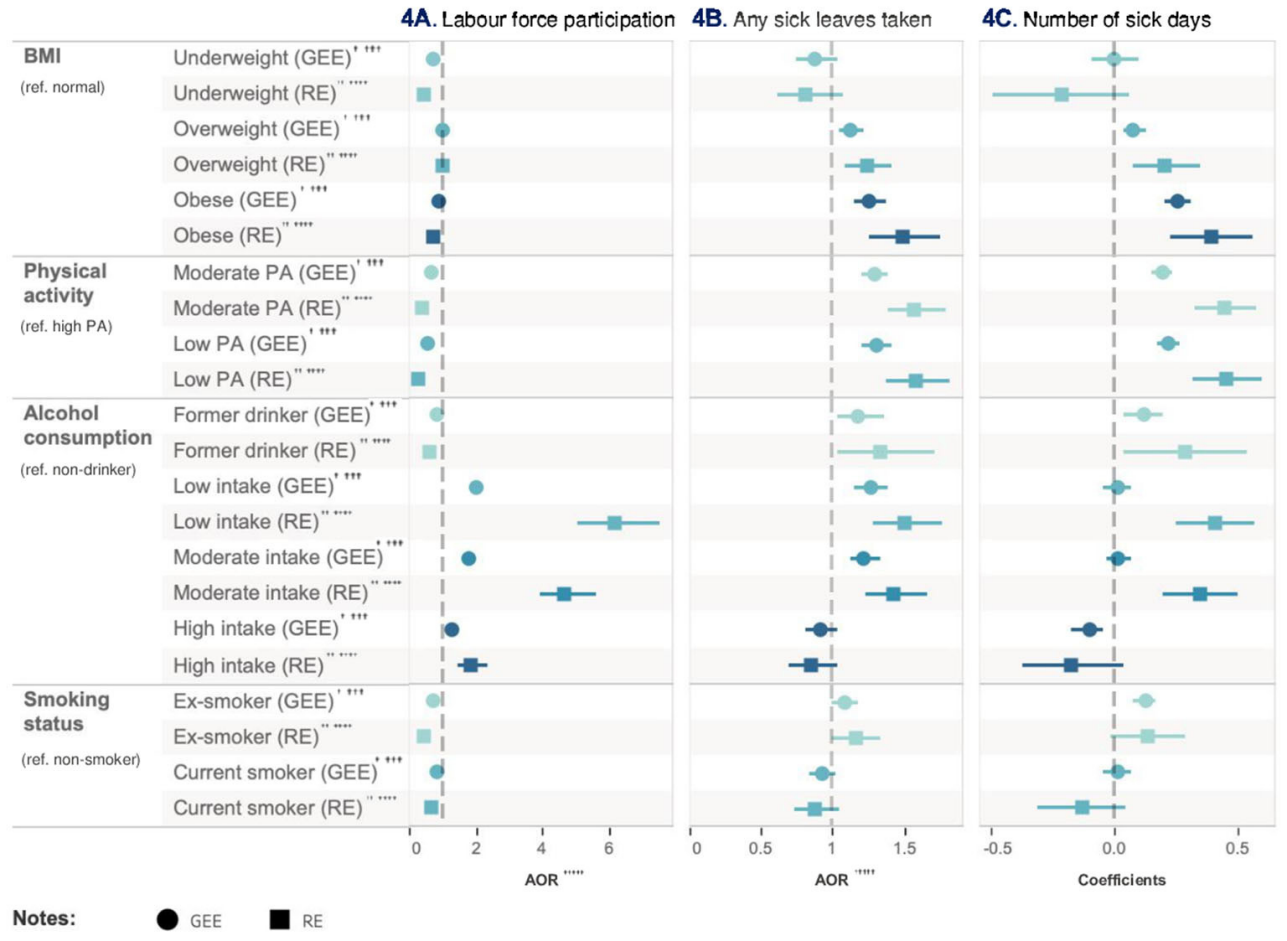
Relative impact of risk factors on HRQoL. ^†^GEE AOR is Model 1: population average model with logit link function. ^††^Random effect (RE) is Model 2: RE logistic model. ^†††^GEE Coefficients is Model 3: population average model. ^††*††*^RE Coefficients is Model 4: is RE linear model. ^††*†††*^AOR is adjusted odds ratio.

In Model 3, obesity (coef = 0.26, 95% CI = 0.21–0.31, *p* < 0.001) had a stronger impact on sick leave days compared with normal BMI after adjusting for all other health risk factors and covariates. Low-level physical activity was the second important risk factor (coef = 0.22, 95% CI = 0.18–0.27, *p* < 0.001). Results from Model 4 were consistent with Model 3, with a relatively higher regression coefficient. Table 5 and Figure 5 show the relative impact of risk factors on work-related outcomes. Table 5a shows the unadjusted model results.

**Table 5:** Relative impact of risk factors on work-related outcomes.

**Risk factors**	**Employment status**		**Any sick leave**		**Number of sick leave days**	
	**Model 1** ^†^	**Model 2** ^††^	**Model 1** ^†^	**Model 2** ^††^	**Model 3** ^†††^	**Model 4** ^††*††*^
	**AOR**^††*†††*^ **(*****P-*****value) (95% CI)**	**AOR**^††*†††*^ **(*****P-*****value) (95% CI)**	**AOR**^††*†††*^ **(*****P-*****value) (95% CI)**	**AOR**^††*†††*^ **(*****P-*****value) (95% CI)**	**Coef**^††*†††*^ **(*****P-*****value) (95% CI)**	**Coef**^††*†††*^ **(*****P-*****value) (95% CI)**
**BMI**						
**Normal (ref)**						
Underweight	0.73 (<0.001) (0.65 to 0.81)	0.43 (<0.001) (0.33 to 0.57)	0.88 (0.120) (0.75 to 1.03)	0.81 (0.131) (0.61 to 1.07)	0.00 (0.968) (−0.09 to 0.10)	−0.21 (0.131) (−0.49 to 0.06)
Overweight	0.10 (0.906) (0.94 to 1.06)	1.00 (0.970) (0.85 to 1.18)	1.13 (0.002) (1.05 to 1.22)	1.24 (0.002) (1.08 to 1.42)	0.08 (0.001) (0.04 to 0.13)	0.21 (0.002) (0.08 to 0.35)
Obese	0.90 (0.003) (0.83 to 0.96)	0.75 (0.005) (0.62 to 0.92)	1.26 (<0.001) (1.15 to 1.38)	1.49 (<0.001) (1.26 to 1.75)	0.26 (0.001) (0.21 to 0.31)	0.40 (<0.001) (0.23 to 0.56)
**Physical activity**						
**High activity (ref)**						
Moderate activity	0.68 (<0.001) (0.64 to 0.73)	0.38 (<0.001) (0.33 to 0.45)	1.30 (<0.001) (1.21 to 1.39)	1.57 (<0.001) (1.39 to 1.79)	0.20 (0.001) (0.15 to 0.24)	0.45 (<0.001) (0.33 to 0.58)
Low activity	0.57 (<0.001) (0.53 to 0.61)	0.23 (<0.001) (0.20 to 0.28)	1.31 (<0.001) (1.21 to 1.41)	1.59 (<0.001) (1.38 to 1.82)	0.22 (0.001) (0.18 to 0.27)	0.46 (<0.001) (0.32 to 0.60)
**Alcohol intake**						
**Non-drinker (ref)**						
No longer drunk	0.83 (<0.001) (0.76 to 0.91)	0.60 (<0.001) (0.47 to 0.76)	1.18 (0.018) (1.03 to 1.36)	1.33 (0.024) (1.04 to 1.71)	0.12 (0.004) (0.04 to 0.20)	0.29 (0.024) (0.04 to 0.54)
Low intake	2.04 (<0.001) (1.88 to 2.20)	6.19 (<0.001) (5.06 to 7.56)	1.27 (<0.001) (1.15 to 1.39)	1.50 (<0.001) (1.28 to 1.77)	0.02 (0.519) (−0.04 to 0.07)	0.41 (<0.001) (0.25 to 0.57)
Moderate intake	1.82 (<0.001) (1.69 to 1.95)	4.70 (<0.001) (3.94 to 5.63)	1.22 (<0.001) (1.12 to 1.33)	1.43 (<0.001) (1.22 to 1.66)	0.02 (0.369) (−0.023 to 0.07)	0.35 (<0.001) (0.20 to 0.50)
High intake	1.28 (<0.001) (1.17 to 1.40)	1.86 (<0.001) (1.47 to 2.35)	0.92 (0.136) (0.81 to 1.02)	0.85 (0.108) (0.69 to 1.04)	−0.10 (0.002) (−0.17 to 0.04)	−0.17 (0.108) (−0.37 to 0.04)
**Smoking status**						
**Non-smoker (ref)**						
Ex-smoker	0.75 (<0.001) (0.69 to 0.80)	0.47 (<0.001) (0.39 to 0.56)	1.08 (0.067) (0.99 to 1.18)	1.16 (0.059) (0.99 to 1.34)	0.13 (0.001) (0.08 to 0.17)	0.14 (0.059) (−0.01 to 0.29)
Current smoker	0.86(0.001) (0.78 to 0.94)	0.67 (<0.001) (0.54 to 0.84)	0.93 (0.137) (0.84 to 1.02)	0.88 (0.147) (0.73 to 1.05)	0.02 (0.525) (−0.04 to 0.07)	−0.13 (0.147) (−0.31 to 0.05)

^†^Model 1 is GEE population average model with logit link function.

^††^Model 2 is random effect logistic model.

^†††^Model 3 is GEE population average model.

^††*††*^Model 4 is random effect linear model.

^††*†††*^AOR, adjusted odds ratio; Coef, is coefficient; 95% C I– 95% confidence interval.

**Table 5a:** Unadjusted regression models for impact of risk factors on work-related outcomes.

**Risk factors**	**Employment status**	**Any sick leave**	**Number of sick leave days**
	**Model 1** ^†^	**Model 2** ^††^	**Model 3** ^†††^
	**AOR**^††*††*^ **(*****P-*****value) (95% CI)**	**AOR**^††*††*^ **(*****P-*****value) (95% CI)**	**Coef**^††*††*^ **(*****P-*****value) (95% CI)**
**BMI**			
**Normal (ref)**			
Underweight	0.68 (<0.001) (0.61 to 0.75)	0.88 (0.094) (0.75 to 1.02)	0.00 (0.982) (−0.09 to 0.09)
Overweight	0.97 (0.413) (0.92 to 1.04)	1.17 (<0.001) (1.07 to 1.26)	0.11 (<0.001) (0.07 to 0.16)
Obese	0.81 (<0.001) (0.75 to 0.87)	1.30 (<0.001) (1.20 to 1.42)	0.31 (<0.001) (0.26 to 0.36)
**Physical activity**			
**High activity (ref)**			
Moderate activity	0.68 (<0.001) (0.64 to 0.72)	1.24 (<0.001) (1.16 to 1.33)	0.19 (<0.001) (0.14 to 0.23)
Low activity	0.55 (<0.001) (0.52 to 0.59)	1.23 (<0.001) (1.14 to 1.32)	0.22 (<0.001) (0.18 to 0.27)
**Alcohol intake**			
**Non-drinker (ref)**			
No longer drunk	0.79 (<0.001) (0.72 to 0.86)	1.13 (0.059) (0.10 to 1.29)	0.13 (0.001) (0.06 to 0.21)
Low intake	2.03 (<0.001) (1.88 to 2.19)	1.29 (<0.001) (1.18 to 1.41)	0.06 (0.020) (0.01 to 0.11)
Moderate intake	1.81 (<0.001) (1.69 to 1.93)	1.27 (<0.001) (1.17 to 1.37)	0.05 (0.030) (0.01 to 0.10)
High intake	1.25 (<0.001) (1.14 to 1.36)	0.97 (0.573) (0.87 to 1.08)	−0.04 (0.170) (−0.10 to 0.02)
**Smoking status**			
**Non-smoker (ref)**			
Ex-smoker	0.78 (<0.001) (0.73 to 0.84)	1.10 (0.014) (1.02 to 1.19)	0.17 (<0.001) (0.12 to 0.21)
Current smoker	0.91 (0.031) (0.83 to 0.99)	0.89 (0.013) (0.81 to 0.97)	0.01 (0.696) (−0.04 to 0.06)

^†^Model 1 is unadjusted GEE population average model with logit link function.

^††^Model 2 is unadjusted GEE population average model.

^††*††*^OR, adjusted odds ratio; Coef, coefficient; 95% C I– 95% confidence interval.

### The relative impact of risk factors on HRQoL

In Model 1, compared with high-level physical activity, engaging in low-level physical activity was associated with a reduced likelihood (AOR = 0.48, 95% CI = 0.44–0.51, *p* < 0.001) of achieving a HRQoL score higher than the population median after adjusting for all other health risk factors and covariates. Similarly, when compared with non-smoker, being a current smoker (AOR = 0.56, 95% CI = 0.52–0.61, *p* < 0.001) and obesity (AOR = 0.63, 95% CI = 0.59–0.68, *p* < 0.001) were associated with a decreased probability of achieving a HRQoL score higher than the population median. Conversely, engaging in low-frequency alcohol intake presents a beneficial impact compared with non-drinker, increasing the likelihood (AOR = 1.37, 95% CI = 1.26–1.49, *p* < 0.001) of achieving a HRQoL score higher than the population median. These findings were consistent in Model 2.

**Figure 6:**
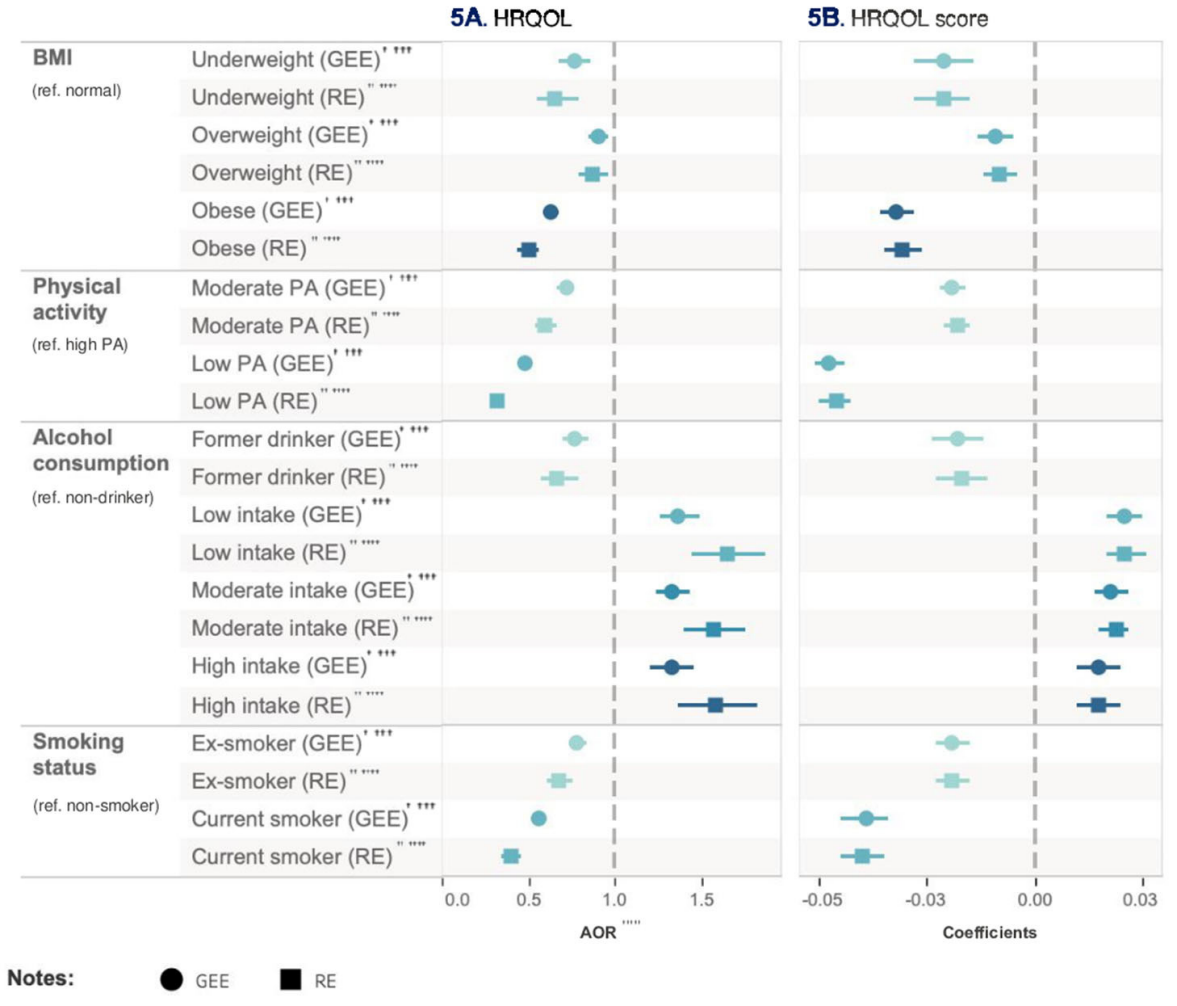
Study flow chart.

In Model 3, the estimated mean HRQoL score for the low-level physical activity group was 0.05 point (95% CI = −0.04 to −0.05, *p* < 0.001) less than high-level physical activity group after adjusting for all other health risk factors and covariates. Conversely, low-frequency alcohol intake was associated with an increase in the mean HRQoL score by 0.02 (95% CI = 0.02–0.03, *p* < 0.001) compared with non-drinker after adjusting for all other health risk factors and covariates. The outcomes from Model 4 were consistent with Model 3. Table 6 and Figure 6 show the relative impact of risk factors on HRQoL. Table 6a shows the unadjusted model results.

**Table 6:** Relative impact of risk factors on HRQoL.

**Risk factors**	**Media HRQoL**		**HRQoL**	
	**Model 1** ^†^	**Model 2** ^††^	**Model 3** ^†††^	**Model 4** ^††*††*^
	**AOR**^††*†††*^ **(*****P-*****value) (95% CI)**	**AOR**^††*†††*^ **(*****P-*****value) (95% CI)**	**Coef**^††*†††*^ **(*****P-*****value) (95% CI)**	**Coef**^††*†††*^ **(*****P-*****value) (95% CI)**
**BMI**				
**Normal (ref)**				
Underweight	0.77 (<0.001) (0.68 to 0.86)	0.66 (<0.001) (0.55 to 0.79)	−0.02 (<0.001) (−0.03 to −0.01)	−0.02 (<0.001) (−0.03 to −0.01)
Overweight	0.91 (0.006) (0.85 to 0.97)	0.87 (0.011) (0.79 to 0.97)	−0.01 (<0.001) (−0.01 to −0.01)	−0.01 (<0.001) (−0.01 to −0.00)
Obese	0.63 (<0.001) (0.59 to 0.68)	0.50 (<0.001) (0.44 to 0.56)	−0.03 (<0.001) (−0.04 to 0.03)	−0.03 (<0.001) (−0.03 to −0.03)
**Physical activity**				
**High activity (ref)**				
Moderate activity	0.72 (<0.001) (0.67 to 0.76)	0.60 (<0.001) (0.54 to 0.67)	−0.02 (<0.001) (−0.02 to −0.02)	−0.02 (<0.001) (−0.02 to −0.01)
Low activity	0.48 (<0.001) (0.44 to 0.51)	0.32 (<0.001) (0.29 to 0.36)	−0.05 (<0.001) (−0.05 to −0.04)	−0.05 (<0.001) (−0.05 to −0.04)
**Alcohol intake**				
**Non-drinker (ref)**				
No longer drunk	0.77 (<0.001) (0.67 to 0.85)	0.67 (<0.001) (0.57 to 0.79)	−0.02 (<0.001) (−0.02 to −0.01)	−0.02 (<0.001) (−0.02 to −0.01)
Low intake	1.37 (<0.001) (1.26 to 1.49)	1.65 (<0.001) (1.45 to 1.87)	0.02 (<0.001) (0.02 to 0.03)	0.02 (<0.001) (0.02 to −0.03)
Moderate intake	1.33 (<0.001) (1.24 to 1.43)	1.57 (<0.001) (1.40 to 1.76)	0.02 (<0.001) (0.01 to 0.02)	0.02 (<0.001) (0.01 to −0.03)
High intake	1.33 (<0.001) (1.21 to 1.46)	1.58 (<0.001) (1.37 to 1.82)	0.01 (<0.001) (0.01 to 0.02)	0.02 (<0.001) (0.01 to 0.02)
**Smoking status**				
**Non-smoker (ref)**				
Ex-smoker	0.78 (<0.001) (0.73 to 0.84)	0.68(<0.001) (0.61 to 0.76)	−0.02 (<0.001) (−0.02 to −0.02)	−0.02 (<0.001) (−0.02 to −0.02)
Current smoker	0.56 (<0.001) (0.52 to 0.61)	0.40(<0.001) (0.35 to 0.46)	−0.04(<0.001) (−0.04 to −0.03)	−0.04 (<0.001) (−0.04 to −0.03)

^†^Model 1 is GEE population average model with logit link function.

^††^Model 2 is random effect logistic model.

^†††^Model 3 is GEE population average model.

^††*††*^Model 4 is random effect linear model.

^††*†††*^AOR, adjusted odds ratio; Coeff, coefficient; 95% CI– 95% confidence interval.

**Table 6a:** Unadjusted regression models for impact of risk factors on HRQoL.

**Risk factors**	**Media HRQoL**	**HRQoL**
	**Model 1** ^†^	**Model 2** ^††^
	**AOR**^†††^ **(*****P-*****value) (95% CI)**	**Coef**^†††^ **(*****P-*****value) (95% CI)**
**BMI**		
**Normal (ref)**		
Underweight	0.68 (<0.001) (0.61 to 0.77)	−0.04 (<0.001) (−0.05 to −0.03)
Overweight	0.89 (<0.001) (0.83 to 0.95)	−0.01 (<0.001) (−0.02 to −0.01)
Obese	0.56 (<0.001) (0.52 to 0.61)	−0.05 (<0.001) (−0.06 to −0.05)
**Physical activity**		
**High activity (ref)**		
Moderate activity	0.71 (<0.001) (0.67 to 0.76)	−0.03 (<0.001) (−0.03 to −0.02)
Low activity	0.45 (<0.001) (0.43 to 0.49)	−0.07 (<0.001) (−0.07 to −0.06)
**Alcohol intake**		
**Non-drinker (ref)**		
No longer drunk	0.72 (<0.001) (0.65 to 0.79)	−0.03 (<0.001) (−0.04 to −0.02)
Low intake	1.40 (<0.001) (1.30 to 1.52)	0.03 (<0.001) (0.02 to 0.04)
Moderate intake	1.34 (<0.001) (1.25 to 1.44)	0.02 (<0.001) (0.02 to 0.03)
High intake	1.29 (<0.001) (1.17 to 1.41)	0.02 (<0.001) (0.01 to 0.02)
**Smoking status**		
**Non-smoker (ref)**		
Ex-smoker	0.77 (<0.001) (0.72 to 0.83)	−0.03 (<0.001) (−0.03 to −0.02)
Current smoker	0.58 (<0.001) (0.54 to 0.63)	−0.05 (<0.001) (−0.06 to −0.04)

^†^Model 1 is GEE population average model with logit link function.

^††^Model 2 is GEE population average model.

^†††^AOR, adjusted odds ratio; Coef, coefficient; 95% CI, 95% confidence interval.

## Discussion

Utilizing data from a comprehensive nationally representative longitudinal survey, this study enriches Australian literature by elucidating the associations between health risk factors and healthcare resource utilization, work-related outcomes, and HRQoL. Insufficient physical activity stands out as the primary risk factor linked to elevated healthcare resource utilization, compromised work-related outcomes and diminished HRQoL. Physical inactivity was associated with a 1.6-fold increase in outpatient visits and a 1.83-fold increase in hospitalisations. Furthermore, it led to a 43% reduction in employment and a 1.31-fold increase in taking sick leave. Individuals with physical inactivity displayed a notable reduction in HRQoL scores, showing a mean decrease of 0.05 in HRQoL. Being ex-smoker emerged as the second predominant risk factor contributing to the health economic burden in this study, as well as impacting work-related outcomes. Individuals who were ex-smokers had a 1.41-fold higher likelihood of outpatient visits and a 1.46-fold increase in hospitalisations compared to non-smokers. Additionally, they experienced a 25% reduction in employment and a 1.16-fold increase in sick leave frequency. Obesity (AOR = 2.12) is the predominant risk factor for medication consumption, whereas physical inactivity typically had the most substantial adverse effect on direct healthcare costs. Low to moderate alcohol consumption has been associated with a modest yet statistically significant decrease in health-care utilization, coupled with enhanced work productivity and health-related quality of life, notwithstanding the observed correlation between alcohol intake and elevated absenteeism due to illness.

The relationship between physical inactivity and health risk factors has been established well (34–43). Eckermann and Willan (44) discovered that physically active individuals have a lower mean utilization in outpatient visits by 2.67 compared to those who are physically inactive. Furthermore, the difference in mean utilization for hospital admissions is reduced by 0.20 (44). Our study aligns with these findings. Another study has discovered that engagement in physical activity among older Australian women results in a reduction of the Odds Ratio (OR) for hospitalization by 0.78 (45). Though the impact of obesity exceeds that of individuals with moderate physical activity, it is imperative to highlight that those with low physical activity levels represent the paramount risk factor for elevated outpatient visits (45). A study from Sweden observed a decrease in hospitalizations attributable to physical inactivity, whereas visits to outpatient clinics due to the same factor sharply escalated by 2016 (46). Concurrently, within a comparable temporal context, our research indicates that the relative impact on outpatient visits and hospitalizations associated with physical inactivity experienced an upsurge. A study investigating the combined effect of physical activity and sickness absence found that inactive smokers accounted for the highest costs associated with short-term sickness absence (47). Although this study aims to quantify individual health risk factors, an exploration into the synergistic effects arising from the coexistence of various health risks within individuals could unveil additional insights, potentially informing and enhancing policy-making strategies.

In relation to smoking-related expenditures, our findings align with recent literature, identifying ex-smokers as an additional risk factor that imposes a burden on both healthcare resources and work-related outcomes (48, 49). Ranabhat et al. (50) emphasized that smoking, sanitation habits and spiritual behaviors could play a role in women's health outcomes. They observed that women who were non-smokers, followed good sanitation practices, and engaged in spiritual activities such as yoga, meditation, and regular exercise had a smaller risk of poor health compared to women who did not engage in these practices (50). This present study showed individuals identified as ex-smokers exhibit a 1.41-fold elevated probability of outpatient visits and a 1.46-fold increase in hospitalisations in comparison to non-smokers. Concurrently, they encountered a 25% reduction in employment and experience a 1.16-fold uptick in the incidence of taking sick leave. Furthermore, ex-smoker was the second most potent risk factor in diminishing HRQoL scores in this study, presenting a decrement of 0.04. Contrarily, two separate case-control studies indicated that current smokers utilized more healthcare services in comparison to ex-smokers (51, 52). This investigation showed that both ex-smokers and current smokers witness a decrease in employment by 25 and 16%, respectively. An additional study implied a potential opportunity for cost savings, approximating $1,135 million AUD in total production, associated with tobacco smoking (35). In our research, being a current smoker emerged as the second greatest risk factor that associated with a reduction in HRQoL scores. Current smokers witnessed a decrease in their mean HRQoL score by 0.04 compared to non-smokers, a less pronounced effect in contrast to ex-smokers. However, an alternate study identified no association between HRQoL scores and smoking status (current or ex-smoker), but rather correlated HRQoL scores with the existence of pulmonary symptoms (53). A recent study discovered that when compared with SF-36, which is one of the commonly used health related measurement tool, SF-6D showed a positive but not statistically significant association on smoking cessation (54). Subsequent studies might investigate the relationship between SF-6D and SF-36 tools in relation to smoking cessation, examining diverse demographic cohorts and utilizing longitudinal designs to comprehend the dynamics of health-related quality of life across various smoking statuses.

Numerous studies have examined the association between obesity and both health resource utilization and productivity loss (55–59). Afshin et al. (59) believed that on a global scale, high BMI was responsible for nearly 40% of all deaths. The health complications linked to a high BMI have been increased (59). High BMI stands out as a principal contributor to years lived with disability worldwide, with significant economic implications for treatment (59). However, insufficient physical activity remains the predominant factor affecting healthcare utilization in the present study.

The relationship between alcohol consumption and healthcare resource utilization is still debatable (14, 60–62). In the current study, alcohol consumption, particularly in low to moderate amounts, demonstrates a protective effect on healthcare resource utilization, while heavy drinking is associated with increased use of health services. Our findings align with current research indicating that alcohol intake has a dose-response effect on health resource consumption, with occasional drinkers presenting a lower risk of utilizing healthcare resource (63). Utilizing a different metric for alcohol consumption in contrast to this study, another research found that individuals reported in low to moderate alcohol consumption displayed a reduced rate of hospitalization and a shorter length of stay, when compared to their heavier-drinking counterparts (64). Although recent research highlights a correlation between excessive alcohol consumption and increased workplace absences (65), this study reveals that even low to moderate drinking were associated with an increase in sick leave utilization. While an increase in sick leave use has been observed among alcohol consumers, this study found an increase in employment associated with alcohol intake, even among heavier drinkers; only former drinkers exhibited a decreased impact on employment. Similar findings were observed in another study, which indicated that individuals who reported abstaining from alcohol seemed to experience a higher prevalence of sickness absence from work compared to participants who drank below a designated risk threshold (66). Results from a previous study indicate that unemployed individuals tend to cease alcohol intake due to “sick quitter effect” (67). Our research corroborates with this conclusion. The variation in outcomes might be attributed to differing definitions of alcohol consumption across various studies. Although previous research has identified a negative correlation between alcohol consumption and quality of life (68), our study reveals that only former drinkers experience lower HRQoL.

## Limitations

This study, however, contains a few limitations. Firstly, our study depended on self-reported data, which is prone to bias, especially in lower socioeconomic groups (52). Secondly, the study employed a short panel data design, limiting our capacity to interpret our findings causally. Thirdly, this study did not explore the impact of diet due to the restricted range of dietary options available in HILDA. Moreover, HILDA has limited representation of unhealthy food choices, such as soft drinks and canned soups, which were not included in the dataset. Fourthly, some of the variables were categorized by frequency. For instance, alcohol intake based on the frequency of consumption, rather than the quantity consumed per day. This may have constrained our ability to capture the association between alcohol consumption and outcome interests, potentially limiting our ability to identify significant trends or patterns in the data. Fifthly, our paper aims to examine the independent effect of a single risk factor on our outcome variables, while controlling for the influence of other potential risk factors, it is possible that each health risk factor may have a combined effect. Future research might consider investigating the synergistic effects of combined risk factors. For instance, the joint impact of smoking and excessive alcohol consumption might prove to be more harmful than the effects of either risk factor in isolation. Additionally, extending the analysis over more than two waves could provide deeper insights into the persistence and magnitude of these associations. Lastly, in addition to traditional risk factors for health such as smoking and overweight, the impact of social and health care perspectives on population health such as political instability, practice of traditional religions, and universal health coverage should be considered (69).

## Conclusions

This study is able to take advantage of the longitudinal database derived from the general population to illustrate health risk factors effect on health resource utilization and work-related outcomes and HRQoL. The utilization of longitudinal data study methodology yields robust estimates regarding the impact of risk factors, thereby enhancing the existing research landscape within Australia. The health risk factors vary in their influence on healthcare utilization, work-related outcomes, and health utility. Our research addresses the current knowledge gap in Australia. We quantify the relationship between the common health risk factors and healthcare utilization, productivity loss, and HRQoL scores. This study underscores that physical inactivity is the predominant risk factor impacting both direct and indirect health costs in Australia. Individuals with obesity may lead to a higher consumption of prescribed medications. Being an ex-smoker emerges as the subsequent significant risk. Hence, public health interventions targeting these risk factors could yield significant benefits in Australia. Conversely, a pattern of low to moderate alcohol consumption appears to exhibit a protective effect on healthcare resource utilization and HRQoL scores. Yet, while alcohol consumption was associated with increased employment, it concurrently elevates instances of absenteeism due to sickness.

## Data availability statement

Requests to access these datasets should be directed to Australian Data Archive Support: ada@anu.edu.au.

## Author contributions

JM: data analysis, interpreting the results, writing the first draft, review and editing. MI: data curation and review. KA: data visualization and review. BM, BF, and AL: review and editing. AT-D: verifying statistical analysis, supervising data interpretation and revision, review, and revising the paper critically for important intellectual content. JL: project lead, conceptualization, supervising, review, and editing.
