# Supplementary material for: Impact of health risk factors on healthcare resource utilization, work-related outcomes and health-related quality of life of Australians: a population-based longitudinal data analysis
Source: Front Public Health. 2023 Nov 27;11:1077793. doi: 10.3389/fpubh.2023.1077793 (PMC10711273; doi:10.3389/fpubh.2023.1077793)
Supplement: Supplementary file 1 [file Table_1.DOCX]

**Supplementary Table S1a: longitudinal data explanatory variables definition**

| Explanatory variables | Definitions |
| --- | --- |
| Smoking status | 1. Non-smoker 2. Ex-smoker 3. Current smoker (including smoke at least weekly, smoke less often than weekly and, smoker daily and above) |
| Alcohol Intake | 1. No alcohol intake is defined as never drink or rarely 2. Former drinker 3. Low-frequency of alcohol intake is defined as 1-2 days per week 4. Moderate-frequency of alcohol intake is defined as 2-4 days per week 5. High-frequency of alcohol intake is defined as defined as 5-6 days or every day per week |
| Physical activity^†^ | 1. High-level physical activity (at least 1500 MET-minutes per week) 2. Moderate-level physical activity (at least 600 MET-minutes per week) 3. Low-level physical activity (not meet any criteria); |
| BMI | 1. Normal BMI (18.5 kg/m^2^-24.9 kg/m^2^) 2. Underweight (<18.5 kg/m^2^) 3. Overweight (25.0 kg/m^2^ – 29.9 kg/m^2^) 4. Obese (30 kg/m^2^ and above) |
| Gender | 1. Male 2. Female |
| Age | 1. 15-24 years old 2. 25-34 years old 3. 35-44 years old 4. 45-54 years old 5. 55-74 years old 6. Age 75-year-old and above |
| Education  Background | 1. Less than senior secondary 2. Secondary school or equivalent   Bachelor or above |
| Employment status | 1. Employed 2. Unemployed |
| Equilibrium household income^†††^ | 1. 1^st^ quintile 2. 2^nd^ quintile 3. 3^rd^ quintile 4. 4^th^ quintile 5. 5^th^ quintile |
| Residency | 1. Urban 2. Rural |
| Marital status | 1. Married or de facto 2. Unmarried 3. Separated, divorced or widowed |
| State | All states are included 0-NSW 1-Vic etc. |
| Country of birth | 1. Australia   1- Main English-speaking countries  2- Others |
| Aboriginal status | 1. Non-indigenous   1- Indigenous |
| Socio-Economic Indexes for Areas (SEIFA)^††††^ | 1. 1^st^ and 2^nd^ decline 2. 3^rd^ and 4^th^ decline 3. 5^th^ and 6^th^ decline 4. 7^th^ and 8^th^ decline 5. 9^th^ and 10^th^ decline |

^†^Physical activity was classified based on International Physical Activity Questionnaire (IPAQ)

^††^BMI was classified based on WHO classification

^†††^Household income was derived from equilibrium family income

^††††^Socioeconomic Indexes for Areas (SEIFA) is developed by Australian Bureau of Statistics that ranks areas in Australia according to relative socio-economic advantage and disadvantage

**Supplementary Table S1b: Outcome variables definition**

| Outcome variables | Definitions |
| --- | --- |
| Any outpatient visit | 1. No outpatient visits in the past 12 months 2. At least one outpatient visits in the past 12 months |
| Number of outpatient visit | Number of outpatient visits in the past 12 months (numeric variable) |
| Any hospitalization | 1. No overnight stay in hospital in the past 12 months 2. At least one overnight stay in hospital in the past 12 months |
| Number of nights in hospital | Number of overnight stays in hospital in the past 12 months (numeric variable) |
| Any medication taken | 1. Not taken any prescribed medication in the past 12 months 2. Taken any prescribed medication in the past 12 months |
| Employment status | 1. Unemployment in last 12 months 2. Employment in last 12 months |
| Any sick leave | 1. no sick leave taken in the past 12 months 2. At least one day sick leave taken in the past 12 months |
| Health-related quality of life | 1. Below median SF-6D scores (median SF-6D score is 0.753) 2. Equal or above median SF-6D scores |

**Supplementary Table S1c: Missing data of explanatory variables**

| Variable name | Missing value |
| --- | --- |
| Sex  Age  Education background  Employment status  Equilibrium household income  Marital status  Residency  State  Country of birth  Aboriginal status  Socio-Economic Indexes for Areas  Smoking status  Alcohol intake  Physical activity  Body Mass Index (BMI) | 0  0  18  0  0  0  4  5  6  14  0  116  2813  309  0 |

**Supplementary Table S2: Relative impact of risk factors on healthcare resource utilisation (outpatient visit)**

| Risk factors | Any outpatient visit | Number of outpatient visit |
| --- | --- | --- |
|  | Model 1  AOR (P value)  95% CI | Model 3  Coef (P value)  95% CI |
| BMI  Normal (ref)  Underweight  Overweight  Obese | 0.95 (0.554) (0.81-1.12)  1.16 (0.001) (1.06-1.27)  1.37(<0.001) (1.23-1.52) | 0.15 (<0.001) (0.09-0.22)  0.08 (<0.001) (0.05-0.12)  0.30 (<0.001) (0.26-0.34) |
| Physical activity  High activity (ref)  Moderate activity  Low activity | 1.49 (<0.001) (1.36-1.63)  1.60 (<0.001) (1.45-1.76) | 0.14 (<0.001) (0.10-0.17)  0.32 (<0.001) (0.28-0.35) |
| Alcohol intake  Non-drinker (ref)  No longer drunk  Low intake  Moderate intake  High intake | 1.24 (0.009) (1.06-1.50)  0.94 (0.235) (0.84-1.04)  0.86 (0.004) (0.78-0.95)  0.92 (0.197) (0.81-1.05) | 0.22 (<0.001) (0.17-0.27)  -0.22 (<0.001) (-0.26- -0.18)  -0.23 (<0.001) (-0.27- -0.19)  -0.25 (<0.001) (-0.30- -0.20) |
| Smoking status  Non-smoker (ref)  Ex-smoker  Current smoker | 1.41 (<0.001) (1.28-1.56)  0.84 (0.001) (0.75-0.93) | 0.20 (<0.001) (0.17-0.24)  0.18 (<0.001) (0.14-0.23) |
| Gender  Male (ref)  Female | 2.22 (<0.001) (2.03-2.43) | 0.31 (<0.001) (0.27-0.34) |
| Age  15-24 (ref)  25-34  35-44  45-54  55-64  65-74  75+ | 1.14 (0.058) (1.00-1.31) 1.13 (0.126) (0.97- 1.32)  1.44 (<0.001) (1.23-1.69)  2.38 (<0.001) (1.99-2.85)  3.71 (<0.001) (2.97-4.64)  6.00 (<0.001) (4.42-8.15) | 0.03 (0.280) (-0.03-0.09)  -0.02 (0.473) (-0.09-0.04)  -0.01 (0.807) (-0.08-0.06)  0.08 (0.020) (0.01-0.15)  0.15 (<0.001) (0.07-0.22)  0.30 (<0.001) (0.21-0.38) |
| Marital status  Married or de facto (ref)  Unmarried  Separated, divorced or widowed | 0.88 (0.028) (0.78-0.99)  0.90 (0.152) (0.78-1.04) | -0.04 (0.132) (-0.08-0.01)  0.06 (0.011) (0.01-0.11) |
| Education  Background  Less than senior secondary (ref)  Secondary school or equivalent  Bachelor or above | 1.24 (<0.001) (1.11-1.38)  1.30 (<0.001) (1.14-1.49) | 0.61 (0.003) (0.02-0.10)  -0.01 (0.697) (-0.06-0.04) |
| Employment status  Employment (ref)  Unemployment | 1.22 (<0.001) (1.10-1.36) | 0.26 (<0.001) (0.22-0.30) |
| Equilibrium household income  1^st^ quintile (ref)  2^nd^ quintile  3^rd^ quintile  4^th^ quintile  5^th^ quintile | 0.93 (0.313) (0.80-1.07)  0.96 (0.561) (0.82-1.11)  1.15 (0.085) (0.98-1.33)  1.09 (0.298) (0.93-1.28) | -0.12 (<0.001) (-0.17- -0.07)  -0.19 (<0.001) (-0.25- -0.13)  -0.15 (<0.001) (-0.21- -0.10) -0.23 (<0.001) (-0.29- -0.17) |
| Residency  Urban (ref)  Rural | 0.87 (0.028) (0.77-0.99) | -0.05 (0.042) (-0.10- -0.00) |
| State  NSW (ref)  VIC  QLD  SA  WA  TAS  NT  ACT | 1.14 (0.029) (1.01-1.28)  0.94 (0.312) (0.84-1.06)  0.95 (0.528) (0.81-1.11)  0.95 (0.513) (0.81-1.11)  0.88 (0.270) (0.70-1.10)  0.64 (0.016) (0.44-0.92)  0.84 (0.200) (0.64-1.10) | 0.03 (0.166) (-0.01-0.08)  -0.00 (0.843) (-0.05-0.04)  -0.04 (0.162) (-0.10-0.02)  -0.02 (0.554) (-0.08-0.04)  -0.09 (0.051) (-0.18-0.00)  -0.18 (0.049) (-0.36- -0.00)  -0.01 (0.922) (-0.12-0.11) |
| Country of birth  Australian (ref)  English-speaking countries  Others | 0.85 (0.035) (0.74-0.99)  0.80 (0.001) (0.70-0.92) | 0.01 (0.674) (-0.04-0.07)  -0.03 (0.336) (-0.08-0.03) |
| Aboriginal status  Non aboriginal (ref)  Aboriginal | 0.95(0.717) (0.712-1.25) | 0.11(0.063) (-0.01-0.22) |
| SEIFA  1^st^ and 2^nd^ decline (ref)  3^rd^ and 4^th^ decline  5^th^ and 6^th^ decline  7^th^ and 8^th^ decline  9^th^ and 10^th^ decline | 0.93 (0.275) (0.81-1.06)  0.99 (0.864) (0.86-1.13)  1.04 (0.617) (0.90-1.19)  0.96 (0.581) (0.83-1.11) | -0.06 (0.018) (-0.12- -0.01)  -0.16 (<0.001) (-0.21- -0.11)  -0.18 (<0.001) (-0.24- -0.13)  -0.22 (<0.001) (-0.27- -0.16) |

^†^Model 1 is GEE population average model with logit link function

^††^ Model 3 is GEE population average model

^†††^AOR - adjusted odds ratio; Coef - coefficient; 95% CI - 95% confidence interval

^††††^NSW-New South Wales; VIC-Victoria; QLD-Queensland; SA-South Australia; W-West Australia; TAS-Tasmania; NT-Northern Territory; ACT-Australian Capital Territory

^†††††^SEIFA -socioeconomic Indexes for Areas

**Supplementary Table S3: Relative impact of risk factors on healthcare resource utilisation (hospitalization)**

| Risk factors | Any hospitalization | Number of hospitalization (in nights) |
| --- | --- | --- |
|  | Model 1  AOR (p value)  95% CI | Model 3  Coef (P value)  95% CI |
| BMI  Normal weight (ref)  Underweight  Overweight  Obese | 1.16 (0.068) (0.99-1.40)  1.09 (0.072) (0.99-1.21)  1.28 (<0.001) (1.15-1.42) | 0.35 (<0.001) (0.27-0.43)  -0.14 (<0.001) (-0.19- -0.09)  0.15 (<0.001) (0.010-0.21) |
| Physical activity  High activity (ref)  Moderate activity  Low activity | 1.28 (<0.001) (1.16-1.41)  1.83 (<0.001) (1.66-2.01) | 0.35 (<0.001) (0.29-0.40)  1.02 (<0.001) (0.97-1.07) |
| Alcohol intake  Non-drinker (ref)  Former drinker  Low intake  Moderate intake  High intake | 1.36 (<0.001) (1.19-1.55)  0.72 (<0.001) (0.64-0.81)  0.75 (<0.001) (0.68-0.83)  0.70 (<0.001) (0.61-0.80) | 0.28 (<0.001) (0.21-0.35)  -0.51 (<0.001) (-0.57- -0.45)  -0.44 (<0.001) (-0.49- -0.38)  -0.44 (<0.001) (-0.51- -0.38) |
| Smoking status  Non-smoker (ref)  Ex-smoker  Current smoker | 1.46 (<0.001) (1.33-1.60)  1.20 (0.002) (1.07-1.35) | 0.59 (<0.001) (0.55-0.64)  0.30 (<0.001) (0.24-0.36) |
| Gender  Male (ref)  Female | 1.21 (<0.001) (1.11-1.32) | 0.21 (<0.001) (0.16-0.261) |
| Age  15-24 (ref)  25-34  35-44  45-54  55-64  65-74  75+ | 1.45 (<0.001) (1.20-1.76)  1.01 (0.946) (0.82-1.24)  0.96 (0.693) (0.77-1.19)  1.14 (0.223) (0.92-1.41)  1.29 (0.024) (1.03-1.62)  1.95 (<0.001) (1.54-2.47) | 0.59 (<0.001) (0.49-0.70)  0.42 (<0.001) (0.31-0.54)  0.65 (<0.001) (0.54-0.76)  0.66 (<0.001) (0.55-0.77)  0.86 (<0.001) (0.75-0.98)  1.17 (<0.001) (1.06-1.31) |
| Marital status  Married or de facto (ref)  Unmarried  Separated, divorced or widowed | 0.67 (<0.001) (0.57-0.78)  1.13 (0.042) (1.00-1.26) | -0.05 (0.195) (-0.12-0.025)  0.25 (<0.001) (0.19-0.31) |
| Education  Background  Less than senior secondary (ref)  Secondary school or equivalent  Bachelor or above | 1.09 (0.090) (0.99-1.22)  1.06 (0.356) (0.93-1.21) | 0.02 (0.414) (-0.03-0.08)  0.18 (<0.001) (0.12-0.25) |
| Employment status  Employment (ref)  Unemployment | 2.00 (<0.001) (1.80-2.23) | 0.99 (<0.001) (0.93-1.04) |
| Equilibrium household income  1^st^ quintile (ref)  2^nd^ quintile  3^rd^ quintile  4^th^ quintile  5^th^ quintile | 0.95 (0.408) (0.83-1.08)  1.17 (0.028) (1.02-1.34)  1.11 (0.143) (0.96-1.29)  1.17 (0.036) (1.01-1.36) | -0.23 (<0.001) (-0.29- -0.16)  0.04 (0.266) (-0.03-0.11)  -0.17 (<0.001) (-0.25- -0.10)  -0.17 (<0.001) (-0.25--0.08) |
| Residency  Urban (ref)  Rural | 1.03 (0.621) (0.91-1.17) | 0.14 (<0.001) (0.08-0.21) |
| State  NSW (ref)  VIC  QLD  SA  WA  TAS  NT  ACT | 1.05 (0.445) (0.93-1.17)  1.11 (0.088) (0.99-1.24)  1.14 (0.091) (0.98-1.33)  1.20 (0.015) (1.04-1.40)  0.81 (0.089) (0.64-1.03)  1.28 (0.301) (0.80-2.03)  1.13 (0.423) (0.84-1.51) | 0.03 (0.356) (-0.03-0.09)  -0.12 (<0.001) (-0.19- -0.06)  -0.17 (<0.001) (-0.26- -0.09)  0.12 (0.004) (0.04-0.20)  -0.33 (<0.001) (-0.46- -0.20)  0.23 (0.084) (-0.03-0.49)  0.25 (0.002) (0.09-0.41) |
| Country of birth  Australian (ref)  English-speaking countries  Others | 0.88 (0.088) (0.77-1.02)  0.72 (<0.001) (0.63-0.83) | 0.07 (0.075) (-0.01-0.14)  -0.40 (<0.001) (-0.47- -0.32) |
| Aboriginal status  Non aboriginal (ref)  Aboriginal | 0.93 (0.615) (0.69-1.24) | -0.16 (0.050) (-0.32- -0.00) |
| SEIFA  1^st^ and 2^nd^ decline (ref)  3^rd^ and 4^th^ decline  5^th^ and 6^th^ decline  7^th^ and 8^th^ decline  9^th^ and 10^th^ decline | 1.00 (0.950) (0.88-1.14)  1.00 (0.969) (0.87-1.14)  0.97 (0.703) (0.85-1.12)  0.95 (0.462) (0.82-1.09) | 0.05 (0.124) (-0.01-0.12)  0.04 (0.293) (-0.03-0.11)  -0.01 (0.743) (-0.08-0.06)  -0.09 (0.023) (-0.17- -0.01) |

^†^Model 1 is GEE population average model with logit link function

^††^ Model 3 is GEE population average model

^†††^AOR - adjusted odds ratio; Coef - coefficient; 95% CI - 95% confidence interval

^††††^NSW-New South Wales; VIC-Victoria; QLD-Queensland; SA-South Australia; W-West Australia; TAS-Tasmania; NT-Northern Territory; ACT-Australian Capital Territory

^†††††^SEIFA -socioeconomic Indexes for Areas

**Supplementary Table S4:** **Relative impact of risk factors on healthcare resource utilisation(medication)**

| Risk factors | | Any medication used |
| --- | --- | --- |
|  |  | Model 1  OR (p value)  95% CI |
| BMI  Normal (ref)  Underweight  Overweight  Obese | | 1.33 (0.001) (1.12-1.57)  1.14 (0.009) (1.03-1.25)  1.49 (<0.000) (1.35-1.65) |
| Physical activity  High activity (ref)  Moderate activity  Low activity | | 1.31 (<0.001) (1.18- 1.44)  1.97 (<0.001) (1.79- 2.17) |
| Alcohol intake  Non-drinker (ref)  No longer drunk  Low intake  Moderate intake  High intake | | 1.50 (<0.001) (1.32-1.71)  0.70 (<0.001) (0.62-0.79)  0.75 (<0.001) (0.68-0.83)  0.74 (<0.001) (0.65-0.84) |
| Smoking status  Non-smoker (ref)  Ex-smoker  Current smoker | | 1.45 (<0.001) (1.33-1.59)  1.19 (0.003) (1.06-1.34) |
| Gender  Male (ref)  Female | 1.20 (<0.001) (1.11-1.30) | |
| Age  15-24 (ref)  25-34  35-44  45-54  55-64  65-74  75+ | 1.44 (<0.001) (1.24-1.67)  2.11 (<0.001) (1.80-2.48)  3.47 (<0.001) (2.97-4.05)  6.57 (<0.001) (5.61-7.69)  10.64 (<0.001) (8.98-12.60)  14.33 (<0.001) (11.76-17.46) | |
| Marital status  Married or de facto (ref)  Unmarried  Separated, divorced or widowed | 1.17 (0.004) (1.05-1.31)  1.17 (0.001) (1.06-1.30) | |
| Education  Background  Less than senior secondary (ref)  Secondary school or equivalent  Bachelor or above | 0.94 (0.178) (0.86-1.03)  0.87 (0.015) (0.78-0.97) | |
| Employment. status  Employment (ref)  Unemployment | 1.43 (<0.001) (1.33-1.55) | |
| Equilibrium household income  1^st^ quintile (ref)  2^nd^ quintile  3^rd^ quintile  4^th^ quintile  5^th^ quintile | 0.86 (0.014) (0.77-0.97)  0.71 (<0.001) (0.63-0.81)  0.75 (<0.001) (0.66-0.86)  0.68 (<0.001) (0.60-0.78) | |
| Residency  Urban (ref)  Rural | 1.00 (0.992) (0.91-1.10) | |
| State  NSW (ref)  VIC  QLD  SA  WA  TAS  NT  ACT | 1.02 (0.669) (0.92-1.13)  0.92 (0.121) (0.83-1.02)  1.07 (0.346) (0.93-1.23)  1.03 (0.723) (0.89-1.18)  1.09 (0.361) (0.90-1.32)  0.73 (0.155) (0.48-1.12)  1.11 (0.443) (0.85-1.44) | |
| Country of birth  Australian (ref)  English-speaking countries  Others | 0.95 (0.410) (0.84-1.07)  0.82 (0.001) (0.73-0.92) | |
| Aboriginal status  Non aboriginal (ref)  Aboriginal | 1.22 (0.119) (0.95-1.56) | |
| SEIFA  1^st^ and 2^nd^ decline (ref)  3^rd^ and 4^th^ decline  5^th^ and 6^th^ decline  7^th^ and 8^th^ decline  9^th^ and 10^th^ decline | 0.87 (<0.001) (0.79-0.97)  0.83 (<0.001) (0.74-0.92)  0.85 (0.005) (0.76-0.95)  0.80 (<0.001) (0.71-0.90) | |

^†^Model 1 is GEE population average model with logit link function

^††^AOR - adjusted odds ratio; 95% CI - 95% confidence interval

^†††^NSW-New South Wales; VIC-Victoria; QLD-Queensland; SA-South Australia; W-West Australia; TAS-Tasmania; NT-Northern Territory; ACT-Australian Capital Territory

^††††^SEIFA -socioeconomic Indexes for Areas

**Supplementary Table S5: Relative impact of risk factors on work-related outcomes**

| Risk factors | Employment | Any sick leave | Number of sick leave (in days) |
| --- | --- | --- | --- |
|  | Model 1  AOR (p value) 95%CI | Model 1  AOR (p value) 95%CI | Model 3  Coef (p value) 95%CI |
| BMI  Normal weight (ref)  Underweight  Overweight  Obese | 0.73 (<0.001) (0.65-0.81)  0.10 (0.906) (0.94-1.06)  0.90 (0.003) (0.83-0.96) | 0.88 (0.120) (0.75-1.03)  1.13 (0.002) (1.05-1.22)  1.26 (<0.001) (1.15-1.38) | 0.00 (0.968) (-0.09-0.10)  0.08 (0.001) (0.04-0.13)  0.26 (0.001) (0.21-0.31) |
| Physical activity  High activity (ref)  Moderate activity  Low activity | 0.68 (<0.001) (0.64-0.73)  0.57 (<0.001) (0.53-0.61) | 1.30 (<0.001) (1.21-1.39)  1.31 (<0.001) (1.21-1.41) | 0.20 (0.001) (0.15-0.24)  0.22 (0.001) (0.18-0.27) |
| Alcohol intake  Non-drinker (ref)  Former drinker  Low intake  Moderate intake  High intake | 0.83 (<0.001) (0.76-0.91)  2.04 (<0.001) (1.88-2.20)  1.82 (<0.001) (1.69-1.95)  1.28 (<0.001) (1.17-1.40) | 1.18 (0.018) (1.03-1.36)  1.27 (<0.001) (1.15-1.39)  1.22 (<0.001) (1.12-1.33)  0.92 (0.136) (0.81-1.02) | 0.12 (0.004) (0.04-0.20)  0.02 (0.519) (-0.04-0.07)  0.02 (0.369) (-0.023-0.07)  -0.10 (0.002) (-0.17-0.04) |
| Smoking status  Non-smoker (ref)  Ex-smoker  Current smoker | 0.75 (<0.001) (0.69-0.80)  0.86 (0.001) (0.78-0.94) | 0.13 (0.001) (0.08-0.17)  0.02 (0.525) (-0.04-0.07) | 0.14 (0.059) (-0.01-0.29)  -0.13 (0.147) (-0.31-0.05) |
| Gender  Male (ref)  Female | 0.59 (<0.001) (0.54-0.64) | 1.18 (<0.001) (1.09-1.28) | 0.10 (<0.001) (0.06-0.15) |
| Age  15-24 (ref)  25-34  35-44  45-54  55-64  65-74  75+ | 1.32 (<0.001) (1.16-1.50)  1.39 (<0.001) (1.21-1.61)  1.69 (<0.001) (1.46-1.95)  0.69 (<0.001) (0.60-0.80)  0.11 (<0.001) (0.09-0.13)  0.03 (<0.001) (0.02-0.03) | 1.95 (<0.001) (1.70-2.23)  1.69 (<0.001) (1.46-1.95)  1.52 (<0.001) (1.31-1.76)  1.34 (<0.001) (1.15-1.57)  0.52 (<0.001) (0.41-0.66)  0.08 (<0.001) (0.03-0.24) | 0.50 (<0.001) (0.42-0.58)  0.45 (<0.001) (0.37-0.54)  0.44 (<0.001) (0.36-0.53)  0.55 (<0.001) (0.47-0.64)  -0.19 (0.005) (-0.32- -0.06)  -2.01 (<0.001) (-2.60- -1.43) |
| Marital status  Married or de facto (ref)  Unmarried  Separated, divorced or widowed | 0.86 (0.004) (0.77-0.95)  1.04 (0.536) (0.92-1.17) | 0.76 (<0.001) 0.68-0.85)  1.11 (0.140) (0.97-1.26) | -0.17 (<0.001) (-0.23- -0.11)  0.17 (<0.001) (0.10-0.24) |
| Education  Background  Less than senior secondary (ref)  Secondary school or equivalent  Bachelor or above | 1.91 (<0.001) (1.74-2.11)  2.70 (<0.001) (2.39-3.06) | 1.38 (<0.001) (1.24-1.55)  1.94 (<0.001) (1.71-2.21 | 0.20 (<0.001) (0.14-0.26)  0.23 (<0.001) (0.16-0.29) |
| Equilibrium household income  1^st^ quintile (ref)  2^nd^ quintile  3^rd^ quintile  4^th^ quintile  5^th^ quintile | 2.54 (<0.001) (2.24-2.89)  3.69 (<0.001) (3.24-4.21)  5.14 (<0.001) (4.47-5.90)  4.25 (<0.001) (3.68-4.90) | 2.02 (<0.001) (1.70-2.41)  2.44 (<0.001) (2.06-2.89)  3.28 (<0.001) (2.78-3.89)  2.60 (<0.001) (2.19-3.10) | 0.34 (<0.001) (0.25-0.44)  0.56 (<0.001) (0.46-0.65)  0.69 (<0.001) (0.60-0.78)  0.59 (<0.001) (0.49-0.68) |
| Residency  Urban (ref)  Rural | 1.00 (0.941) (0.89-1.13) | 0.61 (<0.001) (0.55-0.69) | -0.26 (<0.001) (-0.32- -0.20) |
| State  NSW (ref)  VIC  QLD  SA  WA  TAS  NT  ACT | 1.09 (0.114) (0.98-1.22)  0 .93 (0.198) (0.83-1.04)  1.00 (0.959) (0.87-1.16)  1.08 (0.362) (0.92-1.26)  1.15 (0.226) (0.92-1.43)  2.44 (0.004) (1.32-4.50)  0.94 (0.644) (0.71-1.23) | 1.05 (0.397) (0.94-1.16)  0.94 (0.267) (0.84-1.05)  0.86 (0.041) (0.74-0.99)  0.88 (0.096) (0.76-1.02)  1.00 (0.968) (0.81-1.25)  1.20 (0.344) (0.83-1.73)  1.35 (0.017) (1.05-1.73) | 0.01 (0.765) (-0.05-0.06)  -0.07 (0.012) (-0.13- -0.02)  0.06 (0.113) (-0.01-.014)  -0.02 (0.608) (-0.09-0.05)  -0.14 (0.022) (-0.25- -0.02)  0.22 (0.026) (0.03-0.41)  0.45 (<0.001) (0.33-0.58) |
| Country of birth  Australian (ref)  English-speaking countries  Others | 0.87 (0.057) (0.76-1.00)  0.90 (0.125 (0.79-1.03) | 0.85 (0.027) (0.74--0.92)  0.81 (0.002) (0.71--0.92) | -0.03 (0.428) (-0.10-0.04)  -0.13 (<0.001) (-0.19- -0.06) |
| Aboriginal status  Non aboriginal (ref)  Aboriginal | 0.66 (0.001) (0.52-0.84) | 1.29 (0.082) (0.97-1.72) | 0.08 (0.288) (-0.07-0.23) |
| SEIFA  1^st^ and 2^nd^ decline (ref)  3^rd^ and 4^th^ decline  5^th^ and 6^th^ decline  7^th^ and 8^th^ decline  9^th^ and 10^th^ decline | 1.18 (0.005) (1.05-1.32)  1.26 (<0.001) (1.12-1.42)  1.54 (<0.001) (1.36-1.74)  1.26 (<0.001) (1.11-1.44) | 1.01 (0.931) (0.88-1.14)  0.90 (0.095) (0.79-1.02)  0.87 (0.030) (0.77-0.99)  0.78 (<0.001) (0.68-0.89) | -0.06 (0.105) (-0.12-0.01)  -0.14 (<0.001) (-0.21- -0.07)  -0.13 (<0.001) (-0.20- -0.07)  -0.24 (<0.001) (-0.31- -0.17) |

^†^Model 1 is GEE population average model with logit link function

^††^ Model 3 is GEE population average model

^†††^AOR - adjusted odds ratio; Coef - coefficient; 95% CI - 95% confidence interval

^††††^NSW-New South Wales; VIC-Victoria; QLD-Queensland; SA-South Australia; W-West Australia; TAS-Tasmania; NT-Northern Territory; ACT-Australian Capital Territory

^†††††^SEIFA -socioeconomic Indexes for Areas

**Supplementary Table S6: Relative impact of risk factors on HRQoL**

| Risk factors |  |  |
| --- | --- | --- |
|  | Model 1  AOR (p value)  95% CI | Model 3  Coef (P value)  95% CI |
| BMI  Normal weight (ref)  Underweight  Overweight  Obese | 0.77 (<0.001) (0.68-0.86)  0.91 (0.006) (0.85-0.97)  0.63 (<0.001) (0.59-0.68) | -0.02 (<0.001) (-0.03- -0.01)  -0.01 (<0.001) (-0.01- -0.01)  -0.03 (<0.001) (-0.04-0.03) |
| Physical activity  High activity (ref)  Moderate activity  Low activity | 0.72 (<0.001) (0.67-0.76)  0.48 (<0.001) (0.44-0.51) | -0.02 (<0.001) (-0.02- -0.02)  -0.05 (<0.001) (-0.05- -0.04) |
| Alcohol intake  Non-drinker (ref)  Former drinker  Low intake  Moderate intake  High intake | 0.77 (<0.001) (0.67-0.85)  1.37 (<0.001) (1.26-1.49)  1.33 (<0.001) (1.24-1.43)  1.33 (<0.001) (1.21-1.46) | -0.02 (<0.001) (-0.02- -0.01)  0.02 (<0.001) (0.02-0.03)  0.02 (<0.001) (0.01-0.02)  0.01 (<0.001) (0.01-0.02) |
| Smoking status  Non-smoker (ref)  Ex-smoker  Current smoker | 0.78 (<0.001) (0.73-0.84)  0.56 (<0.001) (0.52-0.61) | -0.02 (<0.001) (-0.02- -0.02)  -0.04 (<0.001) (-0.04--0.03) |
| Gender  Male (ref)  Female | 0.83 (<0.001) (0.77-0.88) | -0.01(<0.001) (-0.02- -0.01) |
| Age  15-24 (ref)  25-34  35-44  45-54  55-64  65-74  75+ | 0.88 (0.040) (0.78-0.99)  0.78 (<0.001) (0.69-0.89)  0.77 (<0.001) (0.67-0.87)  0.75 (<0.001) (0.66-0.86)  0.89 (0.126) (0.77-1.03)  0.65 (<0.001) (0.55-0.77) | -0.02 (<0.001) (-0.02- -0.01)  -0.02 (<0.001) (-0.03- -0.02)  -0.03 (<0.001) (-0.04- -0.02)  -0.03 (<0.001) (-0.04- -0.03)  -0.02 (<0.001) (-0.03- -0.02)  -0.04 (<0.001) (-0.05- -0.03) |
| Marital status  Married or de facto (ref)  Unmarried  Separated, divorced or widowed | 0.72 (<0.001) (0.66-0.80)  0.76 (<0.001) (0.69-0.83) | -0.01 (<0.001) (-0.02- -0.01)  -0.02 (<0.001) (-0.03- -0.01) |
| Education  Background  Less than senior secondary (ref)  Secondary school or equivalent  Bachelor or above | 0.86 (<0.001) (0.79-0.93)  0.86 (0.002) (0.78-0.95) | -0.00 (0.048) (-0.01- -0.00)  -0.00 (0.402) (-0.01-0.00) |
| Employment  Employment (ref)  Unemployment | 0.64 (<0.001) (0.60-0.69) | -0.03 (<0.001) (-0.04- -0.03) |
| Equilibrium household income  1^st^ quintile (ref)  2^nd^ quintile  3^rd^ quintile  4^th^ quintile  5^th^ quintile | 1.35 (<0.001) (1.22-1.50)  1.57 (<0.001) (1.41-1.75)  1.58 (<0.001) (1.41-1.76)  1.73 (<0.001) (1.55-1.95) | 0.02 (<0.001) (0.02-0.03)  0.03 (<0.001) (0.03-0.04)  0.04 (<0.001) (0.03-0.04)  0.04 (<0.001) (0.03-0.05) |
| Residency  Urban (ref)  Rural | 0.95 (0.249) (0.87-1.04) | -0.00 (0.101) (-0.01-0.00) |
| State  NSW (ref)  VIC  QLD  SA  WA  TAS  NT  ACT | 1.05 (0.282) (0.96-1.14)  0.99 (0.823) (0.91-1.08)  0.91 (0.129) (0.81-1.03)  0.94 (0.272) (0.83-1.05)  1.13 (0.187) (0.94-1.34)  1.00 (0.991) (0.72-1.39)  0.86 (0.191) (0.69-1.08) | 0.00 (0.464) (-0.00-0.01)  -0.00 (0.693) (-0.01-0.00)  -0.00 (0.166) (-0.01-0.00)  -0.00 (0.240) (-0.01-0.00)  0.01 (0.052) (-0.00-0.02)  -0.00 (0.692) (-0.02-0.01)  -0.01 (0.104) (-0.02-0.00) |
| Country of birth  Australian (ref)  English-speaking countries  Others | 1.05 (0.362) (0.94-1.17)  0.93 (0.189) (0.84-1.03) | 0.00 (0.284) (-0.00-0.01)  -0.00 (0.191) (-0.01-0.00) |
| Aboriginal status  Non aboriginal (ref)  Aboriginal | 0.95 (0.651) (0.76-1.19) | 0.01 (0.266) (-0.021-0.01) |
| SEIFA  1^st^ and 2^nd^ decline (ref)  3^rd^ and 4^th^ decline  5^th^ and 6^th^ decline  7^th^ and 8^th^ decline  9^th^ and 10^th^ decline | 1.15 (0.003) (1.05-1.27)  1.13 (0.015) (1.02-1.24)  1.21 (<0.001) (1.09-1.33)  1.28 (<0.001) (1.15-1.42) | 0.01 (0.015) (0.00-0.01)  0.01 (<0.001) (0.01-0.02)  0.01 (<0.001) (0.01-0.02)  0.02 (<0.001) (0.01-0.02) |

^†^Model 1 is GEE population average model with logit link function

^††^ Model 3 is GEE population average model

^†††^AOR - adjusted odds ratio; Coef - coefficient; 95% CI - 95% confidence interval

^††††^NSW-New South Wales; VIC-Victoria; QLD-Queensland; SA-South Australia; W-West Australia; TAS-Tasmania; NT-Northern Territory; ACT-Australian Capital Territory

^†††††^SEIFA -socioeconomic Indexes for Areas
